# Size-Fractionated Microbiome Structure in Subarctic Rivers and a Coastal Plume Across DOC and Salinity Gradients

**DOI:** 10.3389/fmicb.2021.760282

**Published:** 2022-01-03

**Authors:** Marie-Amélie Blais, Alex Matveev, Connie Lovejoy, Warwick F. Vincent

**Affiliations:** ^1^Département de Biologie, Institut de Biologie Intégrative et des Systèmes (IBIS) and Takuvik Joint International Laboratory, Université Laval, Quebec City, QC, Canada; ^2^Centre for Northern Studies (CEN), Université Laval, Quebec City, QC, Canada; ^3^Québec-Océan, Université Laval, Quebec City, QC, Canada

**Keywords:** bacteria, microbial eukaryotes, permafrost, river microbiomes, climate change, salinity, dissolved organic carbon, northern ecosystems

## Abstract

Little is known about the microbial diversity of rivers that flow across the changing subarctic landscape. Using amplicon sequencing (rRNA and rRNA genes) combined with HPLC pigment analysis and physicochemical measurements, we investigated the diversity of two size fractions of planktonic Bacteria, Archaea and microbial eukaryotes along environmental gradients in the Great Whale River (GWR), Canada. This large subarctic river drains an extensive watershed that includes areas of thawing permafrost, and discharges into southeastern Hudson Bay as an extensive plume that gradually mixes with the coastal marine waters. The microbial communities differed by size-fraction (separated with a 3-μm filter), and clustered into three distinct environmental groups: (1) the GWR sites throughout a 150-km sampling transect; (2) the GWR plume in Hudson Bay; and (3) small rivers that flow through degraded permafrost landscapes. There was a downstream increase in taxonomic richness along the GWR, suggesting that sub-catchment inputs influence microbial community structure in the absence of sharp environmental gradients. Microbial community structure shifted across the salinity gradient within the plume, with changes in taxonomic composition and diversity. Rivers flowing through degraded permafrost had distinct physicochemical and microbiome characteristics, with allochthonous dissolved organic carbon explaining part of the variation in community structure. Finally, our analyses of the core microbiome indicated that while a substantial part of all communities consisted of generalists, most taxa had a more limited environmental range and may therefore be sensitive to ongoing change.

## Introduction

River ecosystems connect biogeochemical cycles across terrestrial, lacustrine and marine biomes (Aufdenkampe et al., 2011) and are major features of the northern landscape (Vincent and Laybourn-Parry, 2008; Pekel et al., 2016). These northern landscapes are experiencing climate warming and associated transformation through vegetation changes (Jia et al., 2019), hydrological shifts (Wrona et al., 2016) and permafrost degradation (Biskaborn et al., 2019). Rivers act as vectors of these terrestrial changes to the marine environment, and northern seas are highly influenced by their freshwater inflows. The Arctic Ocean receives close to 11% of global river discharge while representing only 1% of the global ocean volume (McClelland et al., 2012), and many biological processes in Arctic marine ecosystems are closely linked to carbon and nutrient inputs from rivers and coastal erosion (Vallières et al., 2008; Terhaar et al., 2021). Additionally, northern rivers are biogeochemical conduits from land to the atmosphere *via* the decomposition of terrestrial carbon and greenhouse gas fluxes across the water–air interface (Kling et al., 1991; Karlsson et al., 2021).

Despite the wide-ranging importance of northern rivers and their biogeochemistry, little is known about their microbiomes, defined as the assemblage of microbial eukaryotes, Bacteria, Archaea and viruses (Grossart et al., 2020). These communities underpin biogeochemical processes and food webs in the aquatic environment, but most analyses of high latitude rivers to date have focused on specific subcomponents. For example, early studies of Archaea in the Mackenzie River, Canada, revealed an unexpected diversity, which was attributed to heterogeneous substrates and to different archaeal populations transported into the river from different parts of the flooded permafrost catchment (Galand et al., 2006). A major transect analysis along 1800 km of the Yenisei River (Russia) showed that bacterial assemblages differed among three sections of the river, and partitioned according to the terrestrial ecozones of mountain taiga, plain taiga and the downstream permafrost region of forest-tundra and tundra (Kolmakova et al., 2014). Bacterial community structure in tundra rivers on Svalbard (Spitzbergen) provided evidence of seasonal changes, attributed in part to increased organic carbon supply in late summer (Kosek et al., 2019). A multi-component analysis of microbiomes along a hydrological continuum (soil water, stream waters, terminal lake) in the Alaskan Arctic tundra showed the effects of dispersal and species sorting, with the downstream diversity of microbial eukaryotes less dependent on dispersal from terrestrial sources than for Bacteria and Archaea (Crump et al., 2012).

The influx of freshwater from rivers into the marine environment creates a transition zone that is characterized by pronounced gradients in chemical properties, including terrestrially derived organic matter, nutrients and salinity (Eyre and Balls, 1999; Dagg et al., 2004; Gebhardt et al., 2004). Salinity is known to act as a major filter in aquatic microbial dispersion, with a controlling effect on the shift in riverine microbiome structure towards brackish and marine taxa (Crump et al., 2004; Lozupone and Knight, 2007; Fortunato and Crump, 2015). Increasing attention has been given to the microbial ecology of these freshwater–saltwater transition zones of northern rivers. In the transition zone of the Mackenzie River, for example, Garneau et al. (2006) found strong gradients in bacterial community structure that correlated with salinity, with a large fraction of the prokaryotic community production associated with particles >3 μm. In a set of transects in Hudson Bay, high-throughput amplicon sequencing showed that colonization of marine coastal waters by freshwater protists was restricted by salinity effects, and that the estuarine communities were a mixture of estuarine specialists, freshwater taxa and marine species (Jacquemot et al., 2021). A study of the prokaryotic and eukaryotic microbiome of coastal lagoons along the eastern Alaskan Beaufort Sea revealed a strong seasonality in community structure, with shifts in energy acquisition pathways associated with seasonal changes in sea ice cover and terrestrial carbon inputs (Kellogg et al., 2019).

Thawing permafrost is likely to affect the microbial community structure of northern rivers and their receiving coastal marine waters in a variety of ways. By increasing hydrological connectivity, permafrost thaw could favor immigration of soil microbes into the river plankton (Crump et al., 2012; Vonk et al., 2015). Degrading permafrost releases soil particles to the aquatic environment, and additional particles may form by the flocculation of dissolved organic matter (DOM); these particles (total suspended sediments, TSS) provide potential substrates for microbial colonization and growth (Deshpande et al., 2016; Cai, 2020). The mobilization of nutrients and organic matter, including dissolved organic carbon (DOC), previously locked within the frozen permafrost changes water geochemistry, hence the resources available to microbes (Frey and McClelland, 2009; Kendrick et al., 2018). Since bacterial taxa differ in their DOM processing capacities, any changes in chemical composition and concentration of DOM are likely to affect bacterial community composition (Amaral et al., 2016; Roiha et al., 2016). Other changes such as nutrient input could increase primary productivity, stimulating other components of the biota, whereas increased DOM and TSS (turbidity) result in decreased light availability in the water column, potentially limiting photosynthesis and favoring heterotrophy.

The various size components of aquatic microbiomes often differ in their species composition and ecology, and therefore size fractionation allows for a more detailed, ecologically relevant analysis. This approach can be used to distinguish picoeukaryotes (cell size 0.22–3 μm) from larger microbial eukaryotes, as well as free-living bacteria from those associated with particles, often assumed to be attached bacteria. These different size-fractions are likely to represent divergent microbial lifestyles and assemblages that play different roles in biogeochemical processes and food webs. Picocyanobacteria and picoeukaryotes may be more efficient in nutrient acquisition and light capture than larger cells in the same phyla, and are more likely to enter the aquatic food web *via* protist grazing (Stockner, 1988). There are pronounced ecological differences among picophytoplankton taxa, however, including in their potential responses to climate change (Flombaum and Martiny, 2021). Particles contain heterogeneous microhabitats, with localized nutrient and organic matter concentrations that may be higher than in the surrounding water (Simon et al., 2002), and they can be important hubs for biogeochemical processes. For example, they contribute to nitrogen loss in the Yangtze and Yellow rivers (China), with low redox conditions in the center of the particles allowing denitrification to occur in oxygenated waters (Xia et al., 2017). Particle-associated communities are often more diverse (Mohit et al., 2014; Bižić-Ionescu et al., 2015; Savio et al., 2015), more metabolically versatile (Lyons and Dobbs, 2012), more productive (Crump et al., 1998; Garneau et al., 2006; Ortega-Retuerta et al., 2013) and taxonomically different (Rieck et al., 2015; Payne et al., 2017; Liu et al., 2020) than their free-living counterparts. However, this is not always the case (Hollibaugh et al., 2000; Ghiglione et al., 2007) and bacteria may also alternate between the two lifestyles (Grossart, 2010).

Our aims in the present study were to characterize the environmental gradients in a set of high latitude rivers, tributaries and coastal receiving waters that are influenced by permafrost degradation; to determine the taxonomic composition and diversity of size-fractionated riverine and coastal microbiomes across these gradients; and to identify relationships between the microbiome variables and potential environmental drivers. Specifically, we focused on the Great Whale River (GWR) region in subarctic Québec, Canada, at the southern limit of permafrost soils where thawing and erosion are proceeding rapidly (Bhiry et al., 2011). The southern part of the catchment lies near James Bay, where the permafrost boundary receded northwards by around 130 km over a recent 50  year period (Thibault and Payette, 2009). The downstream region of the catchment contains eroding permafrost, including a valley sub-catchment containing lithalsa thaw lakes (Bouchard et al., 2014) and an adjacent catchment near the coast of palsa thaw lakes (Arlen-Pouliot and Bhiry, 2005). The GWR discharges into Hudson Bay, and we also examined how the bacterioplankton and microbial eukaryotes differed among size-fractions and shifted along salinity gradients in the river-influenced coastal waters. We hypothesized that variables related to permafrost thaw (TSS, DOM/DOC concentrations, DOM quality, total nutrients) would differ among sub-catchments and cause environmental gradients that act in concert with salinity in the plume to control variations in microbial community structure. We additionally posed the questions: are there systematic changes in alpha and beta diversity across these gradients; is there a core microbiome of prokaryotes and eukaryotes across all environmental conditions; and does the taxonomic makeup and diversity of the microbiome differ between size fractions, including between particle-associated and free-living bacteria.

To address these hypotheses and questions, we sampled a 150-km downstream section of the GWR, along with several of its tributaries, an adjacent river system passing through eroding organic-rich permafrost, and the river plume in Hudson Bay, along its salinity gradient at the mouth of the GWR. Fractionated microbial community composition (0.22–3 μm and 3–30 μm) was determined by high throughput sequencing of the V4 region of 16S and 18S rRNA and rRNA genes. Although our main focus here is on rRNA genes, we used both rRNA and rRNA genes, since the latter can be detected in dead cells, potentially biasing the results, while the former have a higher turnover and can be used as an indicator of living cells (Blazewicz et al., 2013). The concentrations of Bacteria and chlorophyll-containing cells were determined by flow cytometry, and photosynthetic pigments were quantified by high pressure liquid chromatography (HPLC). Colored dissolved organic matter (CDOM) absorbance was used to evaluate DOM quality, and to define the environmental gradients we measured nutrients (total phosphorus, total nitrogen, total dissolved nitrogen), dissolved organic and inorganic carbon, and total suspended sediments.

## Materials and Methods

### Study Sites and Sample Collection

The Great Whale River is 726 km long and discharges into southeastern Hudson Bay near the communities of Whapmagoostui (Cree First Nation) and Kuujjuarapik (Inuit; Commission de toponymie du Gouvernement du Québec, 2021). It is one of the largest rivers of Nunavik (subarctic Québec) and a major freshwater source for the bay, with an annual discharge of 19.77 km^3^ (Déry et al., 2005). The river water creates a large freshwater plume on the surface of the Hudson Bay (Ingram, 1981) and discharges annually 21,000 t of particulate organic matter and 90 × 10^3^ t of dissolved organic carbon to the bay (Hudon et al., 1996). Its watershed of 44,735 km^2^ is predominantly located in a sporadic permafrost area (<2% permafrost coverage) within the lichen woodland zone, except for the coastal area, which is under the climate influence of Hudson Bay and is characterized by discontinuous and scattered permafrost (<50%) and forest-tundra vegetation (Allard and Seguin, 1987; Payette and Rochefort, 2001; Bhiry et al., 2011).

The GWR landscape is heterogeneous and responding to climate change (Allard and Lemay, 2012; Vincent et al., 2017; Kuzyk and Candlish, 2019). The Kwakwatanikapistikw River (hereafter KWK) is a small tributary that enters the GWR approximately 15 km upstream from Hudson Bay. Its valley contains numerous lithalsa thaw lakes (Bouchard et al., 2011), in an area that has recently changed substantially. Between 1959 and 2006, there was a greater than threefold increase in tree vegetation and a near disappearance of permafrost mounds within this lake study area (Bouchard et al., 2014). The nearby Sasapimakwananistikw River (hereafter SAS) flows through another watershed experiencing rapid change and permafrost degradation. This small river passes through a valley of degrading palsa mounds and associated thaw lakes (Arlen-Pouliot and Bhiry, 2005; Figure 7 in Vincent et al., 2017), and discharges into Hudson Bay approximately 1 km southwest of the mouth of the GWR. Additional background information about the GWR and its associated landscapes and marine coastal habitats is given in Nozais et al. (2021).

**Figure 1:**
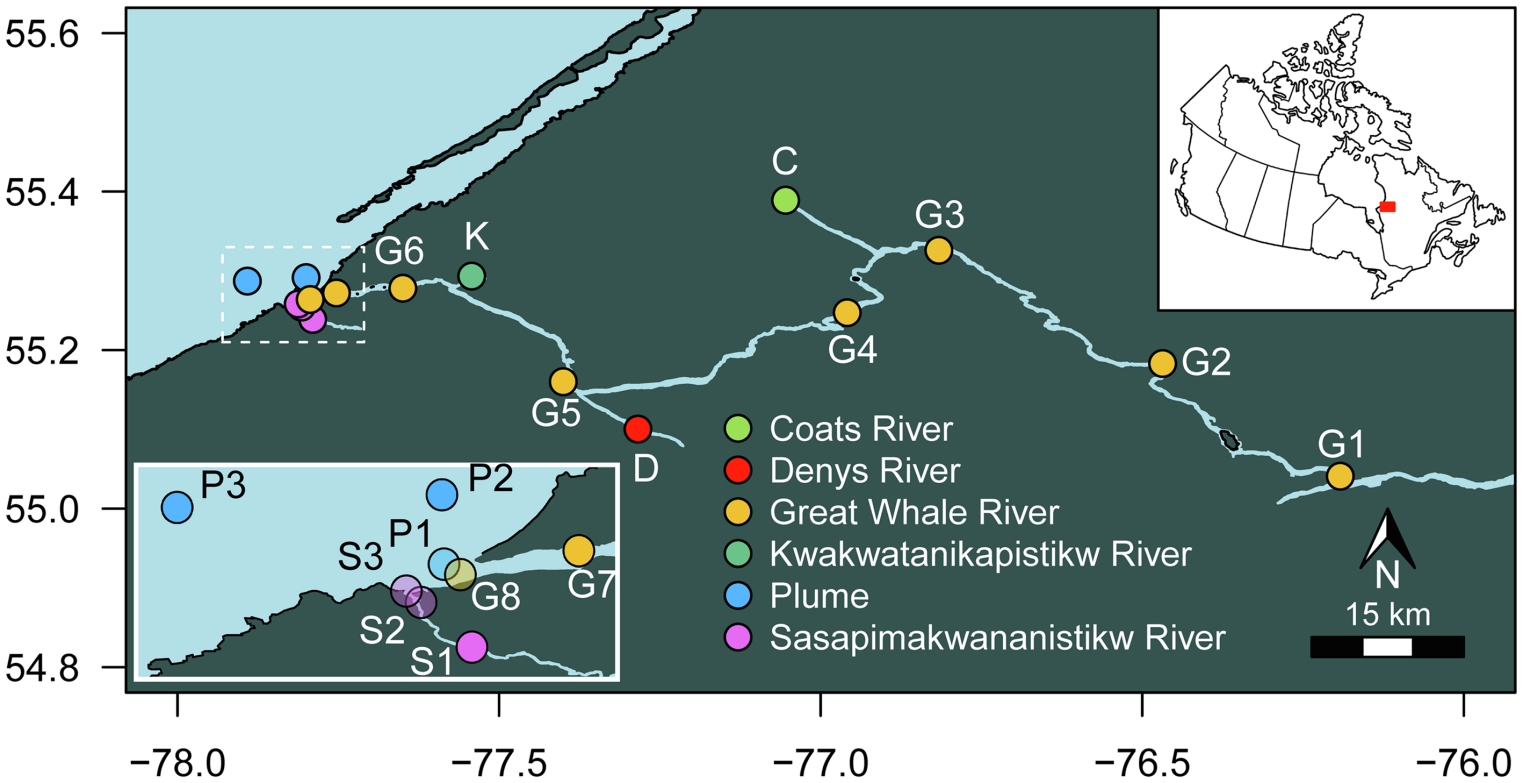
Location of the sampling sites.

The 150-km lower reach of GWR, its tributaries (including KWK, Coats River, and Denys River), SAS River and the plume from GWR flowing into Hudson Bay were sampled from 2 to 10 August 2018 (Figure 1). Sampling in Hudson Bay and at the mouth of SAS and GWR was by boat, and all other sites were from the riverbank, accessed by helicopter. Physicochemical properties were measured *in situ* with an RBR Concerto CTD logger and a Hydrolab DS5X multiparameter probe. Within the plume, the sampling sites were based on the salinity values measured on-site with the Hydrolab. Given the rapid changes of salinity in the plume, conductivity was re-measured in the individual 10 L Cubitainers™ that were used to collect the water samples. For all sites, triplicate surface water samples were collected 5–20 m apart into cleaned Cubitainers™, that were rinsed three times with surface waters and opened submerged 5–10 cm below the surface and then capped. The samples were kept in the dark during transport to the nearby research station in this subarctic region (Center for Northern Studies (CEN), Whapmagoostui-Kuujjuarapik), where they were filtered and subsampled within hours of collection for the molecular, HPLC pigment and water chemistry analyses. The relatively large volume (10 L) would have acted to minimize bottle effects during transport, although the brief delay between collection, filtration or preservation may have allowed some alteration of the microbial communities and chemical parameters. The filtered and preserved samples for chemical measurements were kept in the dark at 4°C until analysis at Laval University and INRS (Quebec City, Canada).

At the subarctic research station, water samples for nucleic acid analysis were prefiltered through a 30-μm mesh to remove zooplankton and then filtered sequentially through a 3-μm pore size, 47-mm diameter polycarbonate filters (large fraction) and 0.22-μm Sterivex™ filter units (Millipore; small fraction) using a peristaltic pump. The volume of water filtered varied (Supplementary Table S1). This serial filtration separated putative free-living from particle-associated bacteria, and for microbial eukaryotes, picophytoplankton were enriched in the smaller size fraction and could be analyzed separately, without dilution by the large fraction. The filters were preserved in RNAlater™ solution (Invitrogen™) before being frozen below −50°C until nucleic acid extraction. Unfiltered water samples (1.8 ml) for flow cytometry (FCM) analysis of bacterial and phytoplankton abundance were transferred to Cryovials and preserved by adding 180 μl of glycerol-TE cryoprotectant solution before being stored frozen below −50°C until analysis.

### Laboratory and Analysis

Water samples for dissolved organic carbon (DOC), total dissolved nitrogen (TDN) and colored dissolved organic matter (CDOM) were filtered through pre-rinsed 0.2-μm cellulose acetate filters (Advantech MFS) and kept in the dark at 4°C in acid-washed glass bottles (60 ml for DOC and TDN, and 120 ml for CDOM). To correct for any DOC released by the cellulose acetate filters, two blanks were run along with samples, after rinsing the filters with ultrapure water, and these blank values (0.12 and 0.16 mg C L^−1^) were subtracted from the DOC results. TDN (detection limit of 0.02 mg N L^−1^) and DOC (detection limit of 0.05 mg C L^−1^) samples were acidified and analyzed by high temperature catalytic oxidation with non-dispersive infrared detection for the DOC (Standard Methods 5310 B) and with chemiluminescence detection for TDN, using a Shimadzu VCPH analyzer. CDOM absorbance was measured between 200 and 800 nm using a Cary 300 Bio UV–Visible Spectrophotometer (Agilent Technologies). All spectra were blank-corrected using ultrapure water and null-point adjustments were made using the mean value from 750 to 800 nm. Absorbance units were converted to absorption coefficients using:


$$a_{\mathrm{CDOM}}\left(\lambda \right)=\frac{2.303\;x\;A\left(\lambda \right)}{L}$$

where *a*_CDOM_(λ) is the absorption coefficient (m^−1^) at wavelength λ, *A*(λ) is the absorbance at that wavelength and *L* is the path length of the optical cell (m). Specific ultraviolet absorbance at 254 nm normalized to DOC (SUVA_254_, L mg C^−1^ m^−1^) was calculated by dividing the UV absorbance (m^−1^) by the DOC concentration (mg L^−1^) and used as proxy for organic matter aromaticity (Weishaar et al., 2003). The absorption coefficient at 320 nm (*a*_320_, m^−1^) was used as indicator of CDOM concentration (Retamal et al., 2007). Spectral slopes (S, Loiselle et al., 2009) for the intervals 279–299 (S_289_), 275–295 (S_285_), 350–400 nm (S_375_) and the spectral slope ratio (S_R_, S_285_/S_375_) were calculated using the abs_parms function of the staRdom package (Pucher et al., 2019) for R. S_R_ was shown to be inversely related to CDOM molecular weight (Helms et al., 2008) and S_289_ is an index of autochthonous carbon (Loiselle et al., 2009; Roiha et al., 2016).

Unfiltered water samples for total phosphorus (TP) and for total nitrogen (TN) were acidified with H_2_SO_4_ (0.1% final concentration) and kept at 4°C in 50 ml Falcon tubes. TP and TN samples were digested with alkaline persulphate and analyzed on a Lachat Autoanalyzer using the ascorbic acid colorimetric method (Standard Methods 4500-P E) for TP (detection limit 5 μg P L^−1^) and the hydrazine reduction followed by sulfanilamide colorimetric method (Standard Methods 12-107-04-1-E) for TN (detection limit 15 μg N L^−1^). Unfiltered water samples for dissolved inorganic carbon (DIC) were acidified (HCl, 0.05 M) and kept in the dark at 4°C in gastight borosilicate glass vials. DIC (detection limit 0.02 mM) was determined with the headspace gas chromatography method using a GC Trace 1310 (Thermo Scientific™). Total suspended sediments (TSS) samples were collected by filtration until clogging, onto a precombusted, preweighed 47-mm GF/F filters (0.7-μm), kept at −60°C at the CEN research station and transferred to −80°C at Laval University (as for pigments, cytometry and amplicon samples), and determined by weighing of dried filters (70°C for 19 h).

Cells for pigment analysis were collected by filtration until clogging onto 0.7-μm GF/F filters, which were stored below −50°C until analysis. The pigments were extracted from the filters with 95% MeOH, analyzed by HPLC as described in Fournier et al. (2021), and attributed to specific taxonomic groups according to Roy et al. (2011). Bacteria and phytoplankton FCM samples were analyzed with a BD Accuri™ C6 flow cytometer (BD Biosciences). For bacteria, 0.5 μl of SYBR Green (1,000X) was added to 200 μl of the sample followed by 15 min incubation at room temperature before being analyzed at a slow flow rate (16 μl min^−1^) for 5 min with the threshold set at 800 for FL1 (green fluorescence). Phytoplankton counts were obtained using their natural fluorescence, with 1 ml of sample analyzed at a fast flow rate (66 μl min^−1^) for 10 min with the threshold set at 800 for FL3 (red fluorescence). Trucount™ tubes (BD Biosciences) were used for the validation and calculation of the flow rate. Fluorescent beads (1 and 3 μm) were used as an internal size standard by adding the beads to GF/F-filtered water samples from each sampling site of different salinities and from the SAS river, with filtered water samples used as blanks.

### Nucleic Acid Extraction

Nucleic acids (RNA and DNA) were extracted from each filter (one large and one small fraction from each site, except for the plume samples and the most upstream site in the GWR (sample G1) for which replicates were extracted and for the plume site at salinity 14.86 for which no filters were extracted). For the extractions, we used the AllPrep DNA/RNA Mini Kit (Qiagen) following a modified version of the manufacturer’s protocol. Briefly, the RNAlater™ solution (Invitrogen™) was removed prior to the extraction and for an optimal cell lysis, lysozyme was added to the mix of Buffer RLT Plus/β-ME before incubation of the filters at 37°C for 45 min. Proteinase K and SDS 10% were then added to the mix and filters were incubated at 65°C for 15 min. A final modification to the protocol was made to ensure the purity of the RNA extract; after the washing of the column with Buffer RW1, DNase (Qiagen RNase-Free DNase set) was added to the RNeasy spin columns (Qiagen) and incubated at room temperature for 15 min. After extraction, the RNA was tested for DNA contamination by polymerase chain reaction (PCR), using one of the DNA extracted samples as positive control. RNA was then converted to cDNA with a High-Capacity cDNA Reverse Transcription Kit (Applied Biosystems).

### Illumina MiSeq Amplicon Library Preparation, Sequencing and Analysis

The microbial community composition of the small and large filtration fractions was determined by amplification and sequencing of the V4 region of the 18S rRNA (cDNA) and 18S rRNA genes (DNA) using primers E572F/E1009R (Comeau et al., 2011) for microbial eukaryotes, and the V4 region of the 16S rRNA (cDNA) and 16S rRNA genes (DNA) using primers 515F (Parada)/806R (Apprill) for Bacteria and Archaea (Apprill et al., 2015; Parada et al., 2016). The amplicon library was prepared by using two single PCR amplification steps, the first to amplify the gene fragment and the second to add Illumina MiSeq adapters and sample indexes. The reaction mix for the first PCR contained 5 μl of Q5® reaction buffer (New England BioLabs), 0.5 μl of Deoxynucleotide Solution Mix (dNTP, New England BioLabs), 0.5 or 1.25 μl of forward and reverse primers, 0.25 μl of Q5® High-Fidelity DNA Polymerase (New England BioLabs), 1, 2 or 3 μl, depending on the concentration, of DNA or cDNA templates and the mix was completed to 25 μl with UltraPure DNase/RNase-Free Distilled Water (Invitrogen™). Successful amplification of some of the extracts required dilution 10 to 50 times to overcome PCR inhibition. PCR conditions for the 18S were 98°C for 30 s; 30 cycles including 98°C for 10 s, 55°C for 30 s and 72°C for 30 s; and finally 4.5 min at 72°C. The PCR conditions for the 16S were 98°C for 30 s; 30 cycles including 98°C for 10 s, 50°C for 30 s and 72°C for 30 s; and finally 5 min at 72°C. The second PCR reactions contained 10 μl of Q5® reaction buffer (New England BioLabs), 1 μl of dNTP (New England BioLabs), 1 μl of each Illumina Index, 0.5 μl of Q5® High-Fidelity DNA Polymerase (New England BioLabs), 1 or 2 μl of PCR 1 product and was completed to 50 μl with UltraPure DNase/RNase-Free Distilled Water (Invitrogen™). The PCR conditions were 98°C for 30 s, 13 cycles including 98°C for 10 s, 55°C for 30 s and 72°C for 30 s; and finally 4.5 min at 72°C. PCR products were verified on a 1% agarose gel and purified using magnetic beads (AMPure XP, Beckman Coulter) after each step. Each PCR included a negative control using the reaction mix without DNA template. Quantification and quality check of the purified second PCR product were done using a Spark® multimode microplate reader (Tecan). PCR products were then pooled equimolarly separately for 18S or 16S, purified and then sequenced on an Illumina MiSeq system, using V3 sequencing chemistry, at the Plateforme d’Analyses Génomiques (IBIS, Laval University, Québec).

Sequences were demultiplexed by the Plateforme d’Analyses Génomiques. Forward and reverse read pairs were merged using BBMerge (v.38.44, Bushnell et al., 2017). Primers were trimmed from the merged reads using fastx_truncate on USEARCH (v.11.0.667, Edgar, 2010), followed by a quality check using FastQC (v.0.11.8, Andrews, 2018) and quality filtering (maxee: 0.5) using fastq_filter on vsearch (v.2.5.0, Rognes et al., 2016). The 16S sequences were quality filtered with Trimmomatic (Bolger et al., 2014) after the removal of primers and before the quality filtering on vsearch. Sequences were dereplicated and sorted using vsearch (v.2.5.0, Rognes et al., 2016), and chimera and singletons were removed from the sequences before being clustered into Operational Taxonomic Units (OTUs) with a similarity threshold of 99% for the eukaryotes and 97% for Bacteria and Archaea, using USEARCH (v.11.0.667, Edgar, 2010). Taxonomy was assigned with the Wang method using mothur (v.1.41.3, Schloss et al., 2009) on the Silva database (v.138, Pruesse et al., 2007; Quast et al., 2013) for Archaea and Bacteria, and the PR2 database (v.4.12.0, Guillou et al., 2013) for microbial eukaryotes. OTUs identified as eukaryotes (204 OTUs), chloroplasts (347 OTUs), domain unknown (379 OTUs) and mitochondria (57 OTUs) were removed from the final 16S OTU table, and those identified as metazoa (55 OTUs) and Embryophyceae (7 OTUs) were removed from the final 18S OTU table. OTUs originating from DNA sequencing are referred to here as rDNA, those from cDNA sequencing as rRNA and those coming from the 0.22-μm and 3-μm filters as small and large, respectively. The sequences reported in this paper have been deposited in NCBI Sequence Read Archive under the BioProject accession number PRJNA744875. OTU tables and fasta files are provided in the Supplementary Material (Supplemental Data Files 1–3).

### Statistical Analysis

All statistical analyses were performed using the software R (v.3.6.3, R Core Team, 2020) in RStudio environment (v.1.2.5001, RStudio Team, 2019). All randomized calculations were made using a set.seed value of 007. Chemical values below the level of detection were considered to be 0 for all statistical analyses. Principal component analysis (PCA) was computed using centered and scaled environmental parameters and was represented using scaling type 1, which preserved the distance between sampling points. Missing values (dissolved oxygen for P2, TSS for G3 and SUVA_254_, *a*_320_ normalized to DOC for P2) were estimated prior to computed to PCA (function imputePCA, missMDA).

The datasets were rarified for the alpha diversity estimates and we note that this alpha diversity underestimates the full diversity in the samples given sequencing bias and the rarefaction itself. All other statistical analysis were performed on the non-rarefied dataset, after confirming that use of this dataset produced the same beta diversity results, as visualized on the NMDS, as using the rarefied dataset. The microbial eukaryote dataset was rarefied to 3,182 reads per sample and bacterioplankton dataset was rarefied to 34,665 reads per sample (with an average of 3,353 and 8,513 total OTUs respectively). The rarefaction and alpha diversity were calculated 100 times using the rarefy_even_depth and estimate_richness function of the phyloseq package (McMurdie and Holmes, 2013) and we report the average diversity estimate. To evaluate relationships between physicochemical parameters or alpha diversity to salinity (in the plume) or physical distance between sampling points (in the GWR), we performed Spearman correlations using the cor.test function. To compare alpha diversity between size fractions, we used Kruskal–Wallis and *post hoc* Dunn’s test with adjusted *p* values using the Benjamini–Hochberg correction (FSA package, Ogle et al., 2020).

Beta diversity indices for the microbial eukaryote and bacterioplankton communities were determined for the rDNA reads using a Bray–Curtis dissimilarity matrix and were visualized on a non-metric multidimensional scaling plot (NMDS). The Bray–Curtis dissimilarity was calculated using proportional abundance of each OTU within each sample. Analysis of similarity (ANOSIM) and a permutational multivariate analysis of variance (ADONIS, 999 permutations) were used to test differences between fractions. Prior to performed ADONIS, the homogeneity of dispersion within a group was evaluated using the vegan betadisper function. Bray–Curtis dissimilarity, ANOSIM and ADONIS were calculated using the vegan package (Oksanen et al., 2019). Bray–Curtis and ANOSIM gave value ranging from 0 to 1, with 0 meaning the same community composition and 1 a completely different community.

The distance–decay relationship in the GWR was determined with a linear regression between the log-transformed (log10) Bray–Curtis values and the distances between pairs of samples. We excluded dissimilarities between G1 triplicates, which were collected within a few meters of each other, to avoid giving weight to small distances in the regression. The regression was performed using the lm function and the assumptions tested using the package gvlma (Peña and Slate, 2006). We calculated the Bray–Curtis dissimilarity on the GWR samples separately for each fraction and nucleic acid. A distance-based redundancy analyses (db-RDA) using the Bray–Curtis dissimilarity matrix was used to quantify the variation in the small and large fraction of the bacterioplankton and the microbial eukaryotes communities (rDNA and rRNA) that could be explained by the physicochemical parameters (capscale function, vegan), followed by a variation partitioning of the significant factors (varpart function, vegan). There was some disparity between the salinity values from *in-situ* probe measurements and those from the Cubitainers™, determined back in the laboratory. We interpreted this as the variability in the plume position with the added complication of boat drift between environmental profiling and sample collection. For this reason, the profiling variables were excluded from the model (temperature, pH and dissolved oxygen). Environmental data were standardized by scaling to zero mean and unit variance (z-score standardization) and a Spearman correlation matrix was examined to reduced multicollinearity (cor function). Selection of explanatory environmental variables was made using a forward selection on adjusted R^2^ values (ordiR2step function, vegan) and the variance inflation factor (VIF) was calculated to verify the collinearity between environmental data in the model. An ANOVA like permutation test (999 permutations) was used to assess the significance of the model. To identify OTUs differing in abundance (p_a_ < 0.01, adjusted using Benjamini–Hochberg correction) between small and large fractions, we performed a differential abundance analysis using the DESeq2 package (Love et al., 2014) and used a script from Monteux et al. (2020) to present the results.

To identify the links between the microbial OTUs and environmental variables, we used a weighted correlation network analysis (WGCNA, Langfelder and Horvath, 2008) as previously described for amplicon datasets (Guidi et al., 2016; Henson et al., 2018; Horton et al., 2019). Prior to the analysis, OTU abundances were Hellinger-transformed and WGCNA was performed separately for the rDNA and rRNA, and for each fraction. A signed scale-free topology adjacency matrix, representing the strength of connection between two OTUs, was created based on OTU pairwise Pearson correlations across all samples raised to a soft power threshold. This adjacency matrix was used to construct a topological overlap matrix for hierarchical clustering analysis to cluster OTUs in modules (subnetworks) of highly connected OTUs. As recommended in the WGCNA tutorial, we merged modules with similar profiles (eigenvalue correlation higher than 0.7) since their OTUs have high co-occurrence. To identify modules most related to environmental variables, each module eigenvalue was pairwise correlated against environmental variables. We selected modules that were significantly and positively correlated with environmental variables and that explained most of the variation in community structure, as determined by the db-RDA, and identified OTUs with a significant (p_a_ < 0.05) positive correlation. All *p*-values were adjusted using the Benjamini–Hochberg correction for multiple correlations (p.adjust function, stats).

## Results

### Environmental Characteristics

**Figure 2:**
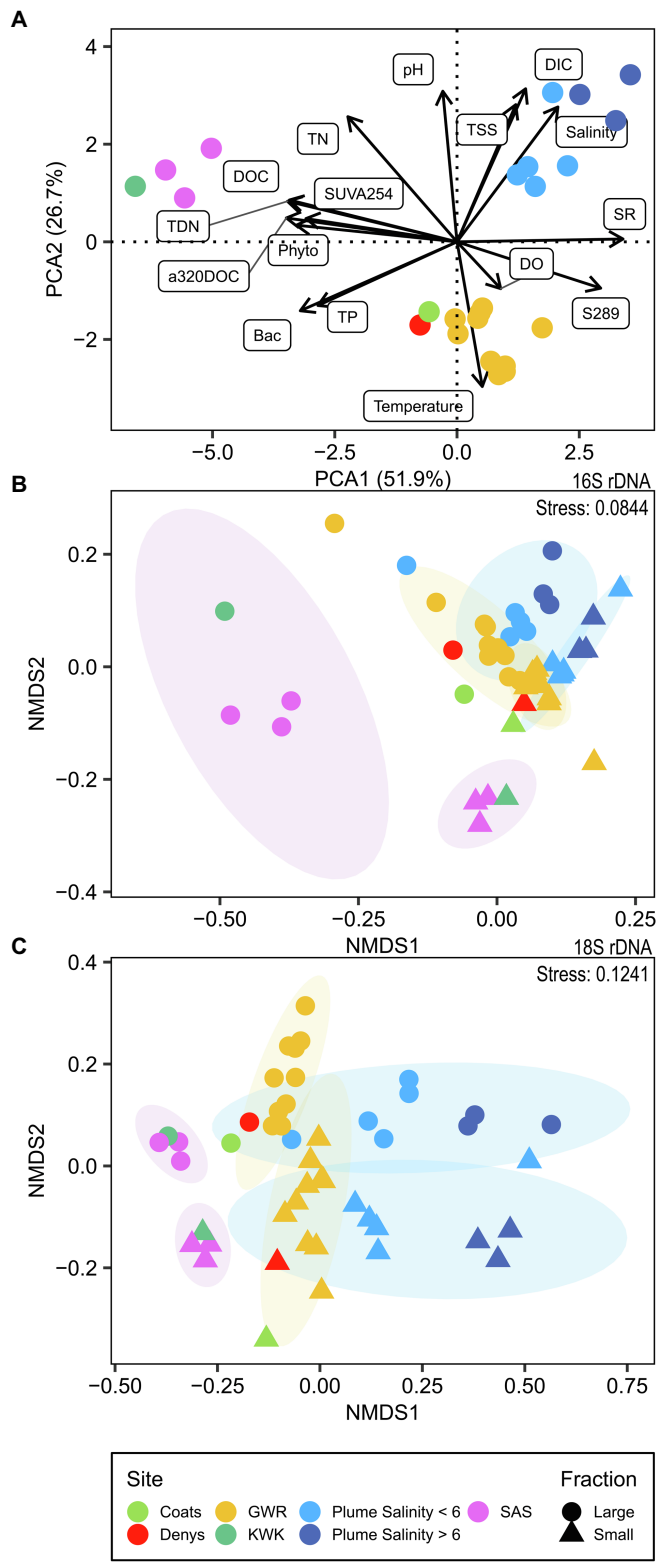
Ordination of environmental variables and microbial communities. **(A)** Principal component analysis, scaling type 1, of standardized physico-chemical parameters; **(B)** NMDS based on Bray–Curtis distance for the bacterioplankton community (16S rDNA); **(C)** NMDS based on Bray–Curtis distance for the microbial eukaryotes (18S rDNA).

The measured physicochemical variables (Supplementary Figure S1) separated the sampling sites into three distinct groups (Figure 2A). The SAS and KWK rivers showed a greater influence of terrestrial organic matter input, as indicated by their high SUVA_254_ values. Their low S_R_ and S_289_ values suggest a higher DOM molecular weight and a smaller contribution of autochthonous DOM. KWK and SAS were also characterized by higher DOC, CDOM (*a*_320_) and nutrients (TP, TN, TDN) than the GWR and its coastal plume, and had higher bacterial and phytoplankton cell concentrations. In SAS, a decrease in concentrations of these variables was observed along the river. Several of these environmental variables were correlated, including DOC with CDOM, nutrients, chlorophyll-*a* and cell concentrations (Supplementary Figure S2).

The plume showed the effects of mixing of freshwater from GWR with seawater in the bay (Supplementary Figure S3). These coastal waters had the highest DIC concentrations of all samples analyzed, with salinity values that ranged from 1.16 to 14.86. The plume samples had higher TN, but lower TP and bacterial cell concentrations than the GWR. CDOM concentrations correlated negatively with the salinity, while its molecular weight (as indicated by S_R_ values) correlated positively (ρ = −0.93, *p* < 0.001, and ρ = 0.80, *p* = 0.01, respectively; Supplementary Figure S4). There were higher concentrations of suspended sediments in the plume, and pH increased with increasing salinity.

An increase in TSS concentrations was observed with distance downstream in the GWR, while there was little change in DIC, DOC, CDOM, nutrients and bacterial cell concentrations throughout the 150 km freshwater reach of the river. Phytoplankton cell concentrations in the GWR increased after the confluence of the Coats and Denys rivers and increased over the sampled reach of the river by a factor of two. The environmental characteristics of Coats and Denys rivers were similar to GWR except for higher DOC, chlorophyll-*a* and phytoplankton cell concentrations, and lower bacterial cell concentrations. Values of pH increased along the SAS river and the GWR until near Kuujjuarapik-Whapmagoostui (G7 and G8), where a decrease was observed. Oxygen concentrations were generally near or slightly above saturation at all sampled stations, except for the SAS mouth and site P3 in the plume (Supplementary Table S2).

### Phototrophic Pigment Composition

The HPLC analyses showed that a broad range of pigments were present at all stations (Supplementary Figures S5, S6), specifically (in addition to chlorophyll-*a*): chlorophyll-*b* and -*c* (*c_1_* and *c_2_*), Mg 2,4-divinylpheoporphyrin (MgDVP), β,Ɛ-carotene, β,β-carotene, peridinin, fucoxanthin, 9-cis-neoxanthin, violaxanthin, diadinoxanthin, antheraxanthin, alloxanthin, zeaxanthin, and lutein with trace levels also of echinenone in many samples. The dinoflagellate pigment peridinin, and the cryptophyte pigments crocoxanthin and alloxanthin were conspicuously higher (per unit chlorophyll-*a*; concentration ratio) in the plume at intermediate salinities (salinities 7.59, 8.39, 10.07; site P2). Lutein (green algae) and zeaxanthin (green algae and cyanobacteria) occurred in peak concentrations at the KWK river station, while the SAS samples had higher concentrations of violaxanthin (green algae but also other groups) compared to other sites. Fucoxanthin dropped to low values with an increase in salinity up to 10.07, rose again at salinity 14.86, and was also at high concentrations in the Coats River. Violaxanthin and lutein decreased with increasing salinity. The freshwater cyanobacterial pigment canthaxanthin showed a general trend of increasing concentration down the GWR to a maximum at G3, but was absent from the plume at salinities higher than 5.09. Similarly, the related pigment astaxanthin occurred throughout the GWR, but was absent from the plume and the SAS River. In general, highest concentrations of photoprotective carotenoids and photosynthetic pigments (including chlorophyll-*a*) occurred in the SAS and KWK rivers. The full HPLC pigment data set is archived in Blais et al. (2021).

### Microbial Community Composition and Diversity

**Figure 3:**
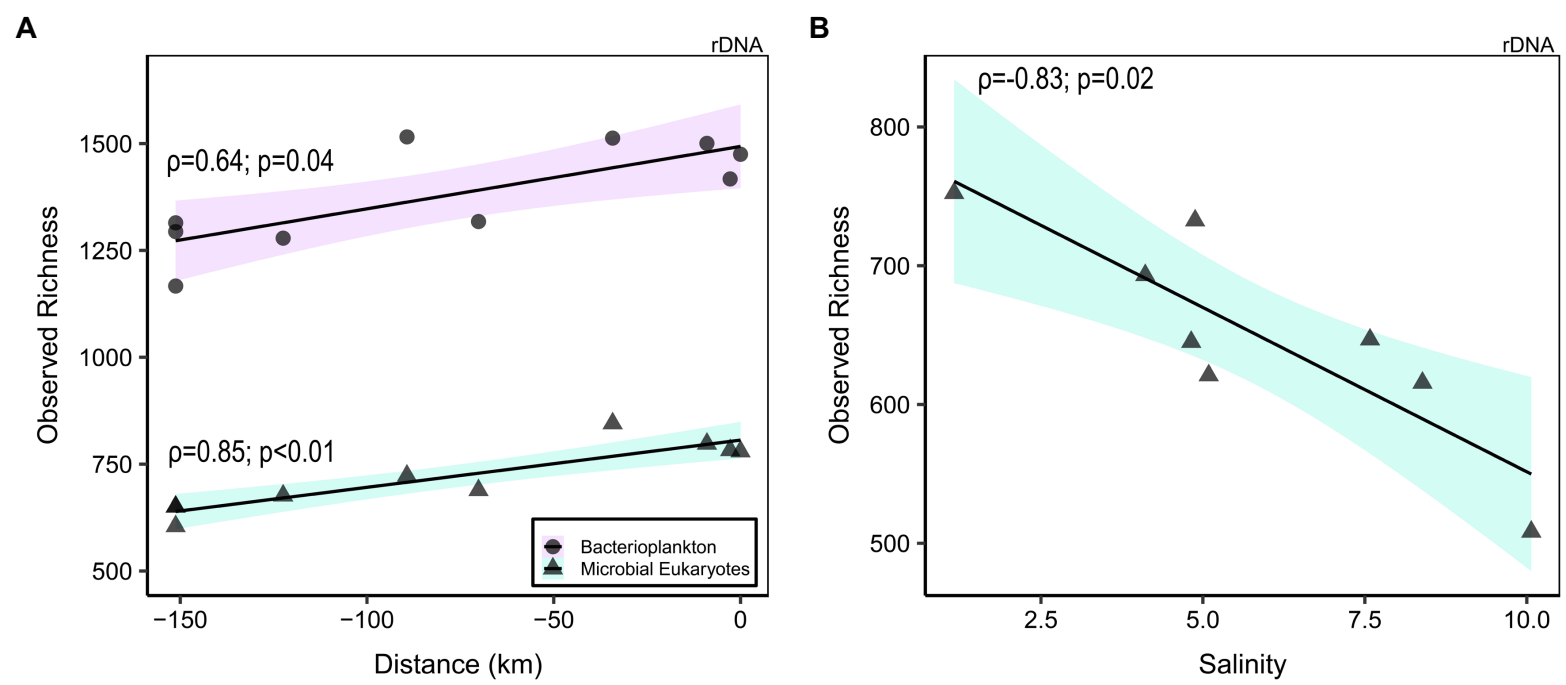
Richness of bacterioplankton (rDNA small fraction; circles) and microbial eukaryotes (rDNA large fraction; triangles) in the Great Whale River as a function of distance downstream (**(A)**; 0 km = river mouth) and salinity in the plume **(B)**. Linear regression was plotted to highlight the correlation.

After the quality filtering, we retained 11,696,390 reads (121,837 ± 48,965, mean ± SD, reads per sample; *n* = 96) for bacterioplankton (here including Archaea and Bacteria) and 2,312,415 reads (22,213 ± 12,774; *n* = 96) for microscopic eukaryotes and fungi (collectively referred to as microbial eukaryotes), which clustered into 8,673 Bacteria and Archaea and 3,383 eukaryote OTUs, respectively. The observed richness values (number of OTUs) were typically at least two times greater for the bacterioplankton than the microbial eukaryotes (Supplementary Figure S7; Supplementary Tables S3 and S4). The rDNA bacterioplankton richness of the large fraction was systematically greater than in the small fraction (*p* < 0.01, Kruskal–Wallis), and the small fraction diversity increased along the GWR (rDNA: ρ = 0.64, *p* = 0.04, not significant for rRNA; Figure 3A). For the microbial eukaryotes, the small fraction richness was greater for SAS and KWK rivers (*p* = 0.02), but no consistent trend was observed in the plume nor in the GWR. The large fraction richness increased along the GWR (ρ = 0.85, *p* < 0.01; Figure 3A) and decreased with the salinity in the plume (ρ = −0.83, *p* = 0.02; Figure 3B); these trends were similar for the rRNA but not significant.

Archaea accounted for less than 0.08% of the reads for each sample, with a total of 94 OTUs. These were mainly dominated by the Order Woesearchaeales, with a markedly higher proportion in the SAS river (Supplementary Figure S8). The archaeal reads included the nitrifier *Nitrosopumilus* and methanogens such as *Methanoregula*, *Methanosaeta* and *Methanobacterium*.

The bacterial community was composed of 51 phyla, of which 22 each accounted for more than 0.1% of the reads (Supplementary Figure S9). Bacterial communities (both rRNA and rDNA) were predominantly composed of Proteobacteria (mostly Burkholderiaceae and Comamonadaceae), Actinobacteriota (mostly Sporichthyaceae), Bacteroidota (previously known as Bacteroidetes; mostly Chitinophagaceae) and Verrucomicrobiota (mostly Pedosphaeraceae). The relative abundance of Actinobacteriota was consistently lower in rRNA, potentially due to their low growth rates. Cyanobacteria contributed less than 0.5% of the bacterial reads at most sites, but contained diverse taxa including the picocyanobacteria *Cyanobium* and *Synechococcus*, filamentous oscillatorians such as *Pseudanabaena*, *Tychonema* and *Leptolyngbya*, colonial taxa such as *Snowella*, and nitrogen fixing genera such as *Aphanizomenon*, *Anabaena* (*Dolichospermum*), *Nostoc*, *Rivularia* and *Calothrix* (Supplementary Figure S10).

The microbial eukaryote communities (both rRNA and rDNA) were composed of nine major taxonomic groups dominated by Alveolata (mostly Dinoflagellata, Ciliphora and *Perkinsea*) and Stramenopiles (mostly Ochrophyta) and to a lesser extent Hacrobia (mostly Cryptophyta, Centroheliozoa and Katablepharidophyta). In addition, there were representatives of Opisthokonta (mostly Choanoflagellida), Rhizaria (mostly Cercozoa) and Archaeplastida (mostly Chlorophyta; Supplementary Figure S11). OTUs unique to the plume included the Mamiellales picochlorophytes *Bathycoccus prasinos* and *Micromonas* (clade B3), and the diatom *Skeletonema*.

### Core Microbiome

**Figure 4:**
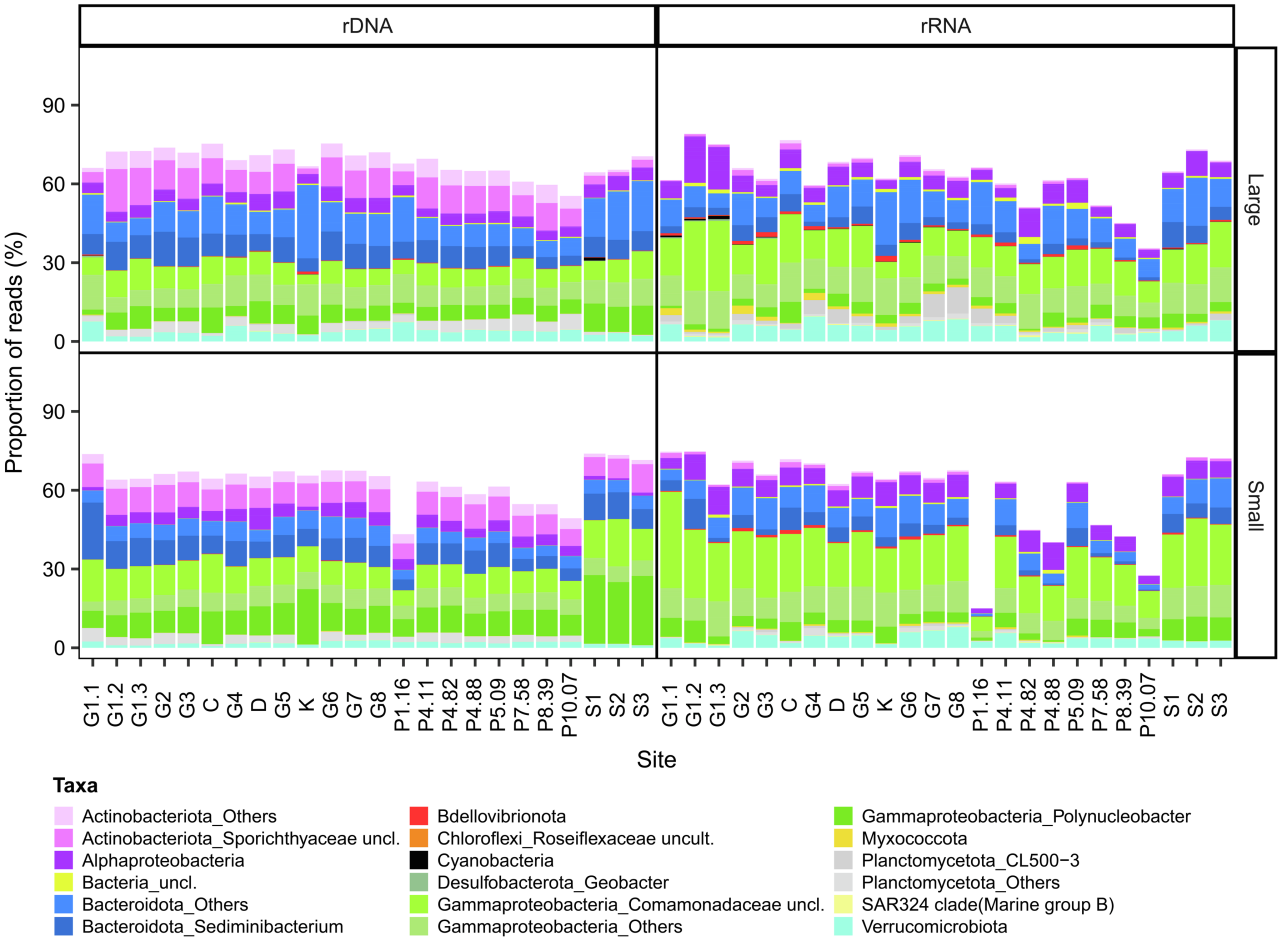
Bacterial composition of the core microbiome in GWR and associated waters. The values are % reads for 16S rDNA or rRNA in the large **(top)** and small **(bottom)** fractions.

The bacterial component of the core microbiome was defined as OTUs present in both the rRNA and the rDNA reads at all sites (Figure 4, Core OTU tables are available in the Supplementary Data File 3 as Supplementary Tables S5–S8). This consisted of 198 OTUs for the small fraction (representing 15–77% of the reads and 2.42% of the total OTUs of this fraction) and 322 OTUs for the large fraction (36–79% of the reads and 3.77% of the total OTUs of this fraction). This core microbiome was represented by 11 phyla: Proteobacteria, Bacteroidota, Actinobacteriota, Bdellovibrionota, Myxococcota, Planctomycetota, SAR324 clade (Marine group B), Verrucomicrobiota for both fractions and Cyanobacteria (*Pseudanabaena PCC-7429* and *Snowella*), Desulfobacterota, Chloroflexi, for the large fraction. The core was predominantly represented by *Polynucleobacter*, *Sediminibacterium* (Chitinophagaceae), unclassified Sporichthyaceae, unclassified Comamonadaceae and the NS11-12 marine group.

**Figure 5:**
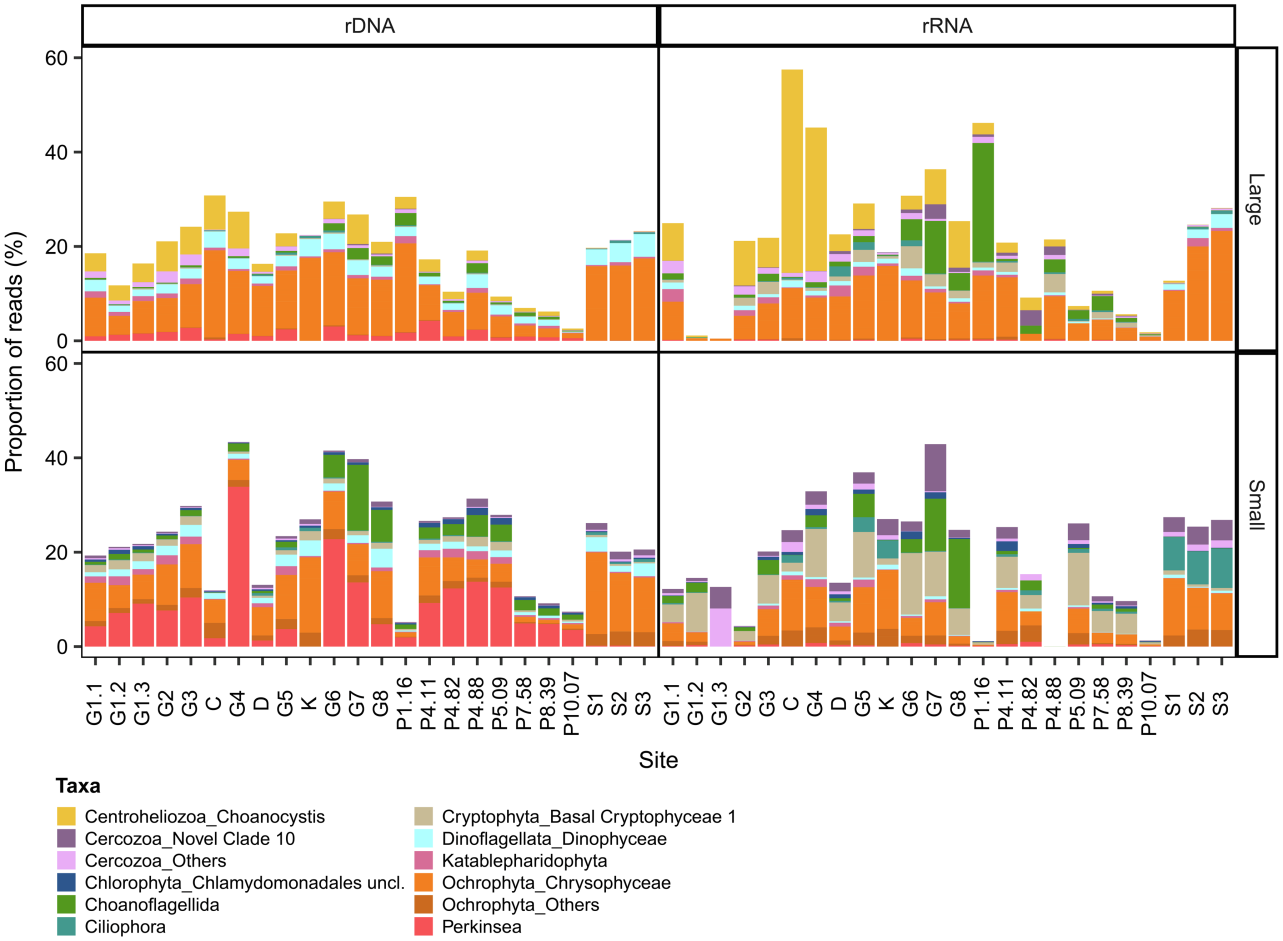
Eukaryotic composition of the core microbiome in GWR and associated waters. The values are % reads for 18S rDNA or rRNA in the large **(top)** and small **(bottom)** fractions.

The microbial eukaryote communities showed much greater variability in OTU composition, and our bacterial definition of a core microbiome could not be applied. This variability included the over-representation of Ciliophora for some rRNA samples, and high read proportions for unclassified eukaryotes in the Coats River (rDNA, small) and in some GWR samples (G6 rRNA, small, G8 rRNA small and large). To adjust for these outliers, we therefore defined the microbial eukaryote core microbiome as OTUs present in the both the rRNA and the rDNA reads in 90% of the samples. The microbial eukaryote core microbiome was classified into six broad groups (Alveolata, Archeaplastida, Opisthokonta, Rhizaria, Stramenopiles, and the polyphyletic Hacrobia) and consisted of 57 OTUs for the small fraction (0.01–43% of the reads and 1.74% of the total OTUs of this fraction) and 48 OTUs for the large fraction (0.47–57% of the reads and 1.45% of the total OTUs of this fraction; Figure 5). In the plume, the proportion of core reads generally decreased with increasing salinity. The most abundant OTUs were members of the class Perkinsida and Chrysophyceae in the small fraction, and Chrysophyceae and the genus *Choanocystis* in the large.

### Beta Diversity and Distance–Decay Relationship

Beta diversity of the rDNA OTUs revealed a weak separation between small and large fractions of the bacterioplankton and microbial eukaryote communities (ADONIS *p* < 0.001 for both and ANOSIM *R* = 0.2754, *p* = 0.001 for bacterioplankton and *R* = 0.3021, *p* = 0.001 for microbial eukaryotes). There was clustering of beta diversity among sites (Figures 2B, 2C). The GWR communities tended to cluster together, as did the plume communities where they were more similar within the same salinity range, except for the lowest salinity (1.16) communities where the large fraction clustered with the GWR and the small fraction was apart from the others. SAS and KWK communities clustered, together except for the large fraction of the bacterioplankton community, while communities from Coats and Denys rivers clustered between GWR and SAS/KWK.

Distance–decay relationships based on the transformed Bray–Curtis dissimilarity (log10) and the distance between the sampling points of the GWR were investigated with linear models. Both of the rDNA fractions for microbial eukaryote communities (rDNA large fraction R^2^ = 0.63, *p* < 0.001; small fraction R^2^ = 0.34, *p* < 0.001; Supplementary Figure S12) showed a significant distance–decay relationship, meaning that community dissimilarity increased with increasing distance between sampling points. The microbial eukaryote rRNA showed a similar relationship, but the high proportion of Ciliophora reads (especially in the large fraction) had a strong influence. As for bacterioplankton, the distance–decay relationship was significant for the rDNA large fraction (R^2^ = 0.16, *p* = 0.008), however, the linear regression assumptions were not satisfied for the rDNA small fraction, mainly because of the sampling site G1.1 that had a higher dissimilarity irrespective of distance to other sites. For the rRNA sequences, the distance–decay relationship was significant for both fractions (rRNA small fraction R^2^ = 0.28, *p* < 0.001 and large fraction R^2^ = 0.32, *p* < 0.001; Supplementary Figure S13).

### Differences Between Fractions

**Figure 6:**
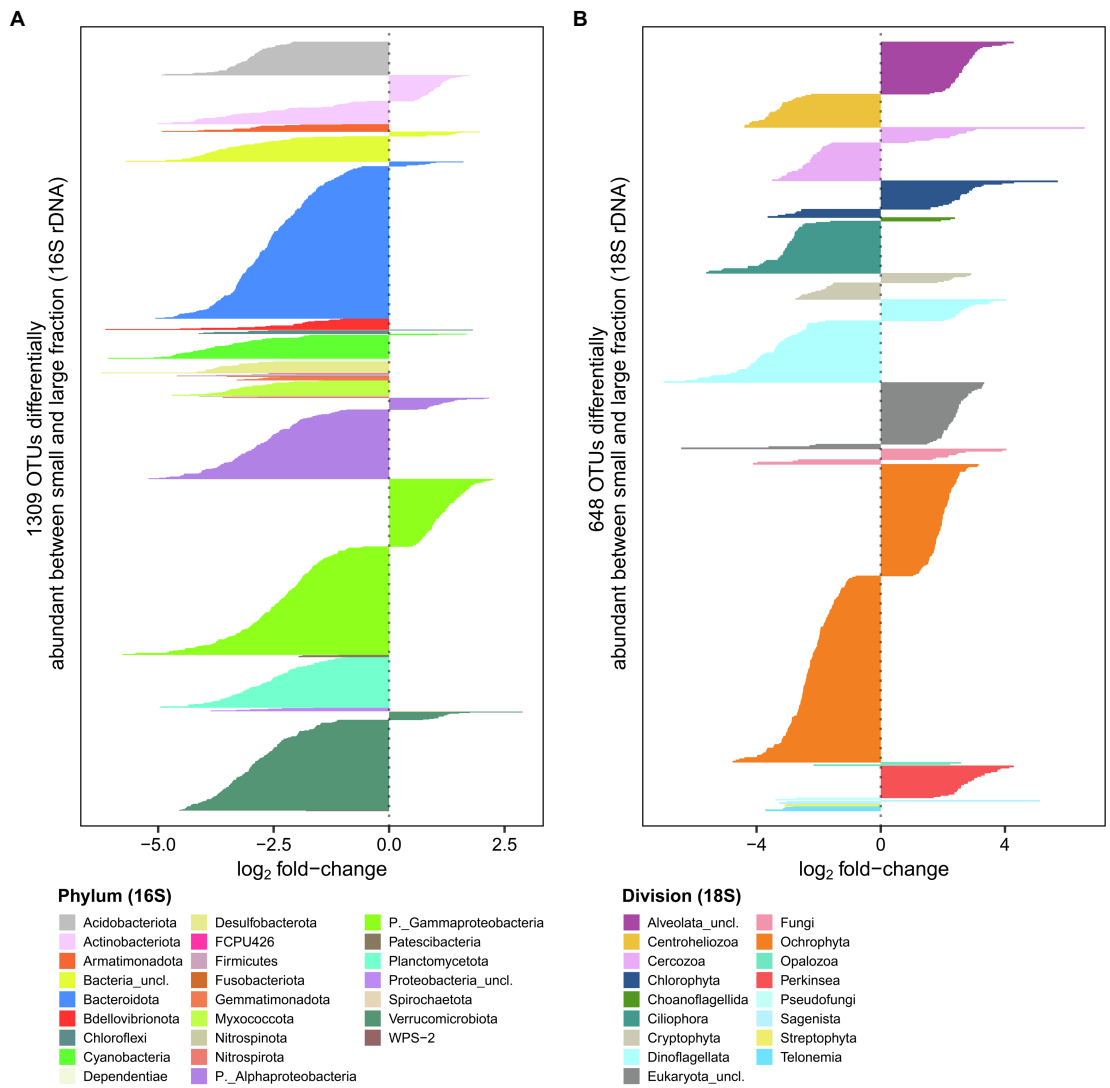
Comparison of large and small fractions for bacterioplankton **(A)** and microbial eukaryotes **(B)**. Negative values represent rDNA OTUs more abundant in the large fraction, while positive values represent OTUs more abundant the small fraction.

To evaluate differences between size fractions, we performed a differential abundance analysis on the rDNA sequences. There were 1,309 OTUs (16% of rDNA OTUs) that were significantly different between fractions for the bacterioplankton and 648 OTUs (20% of rDNA OTUs) for the microbial eukaryotes (Figure 6). The bacterioplankton OTUs that were differentially abundant were mainly enriched in the large fraction, often to a substantial extent. Phyla enriched in the large fraction included Acidobacteriota, Armatimonadota, Bdellovibrionota, Desulfobacterota and Planctomycetota, while unclassified Burkholderiales (Gammaproteobacteria) had a higher number of OTUs enriched in the small fraction. For the microbial eukaryotes, OTUs with the greatest differences between fractions were mainly from the Centroheliozoa, Ciliophora, Dinoflagellata and Ochrophyta taxa, which were more represented in the large fraction, and *Perkinsea*, Chlorophyta, Dinoflagellata (*Prorocentrum*) and Cercozoa (Filosa-Thecofilosea) in the small fraction.

### Environmental Drivers of Microbial Community Structure

**Figure 7:**
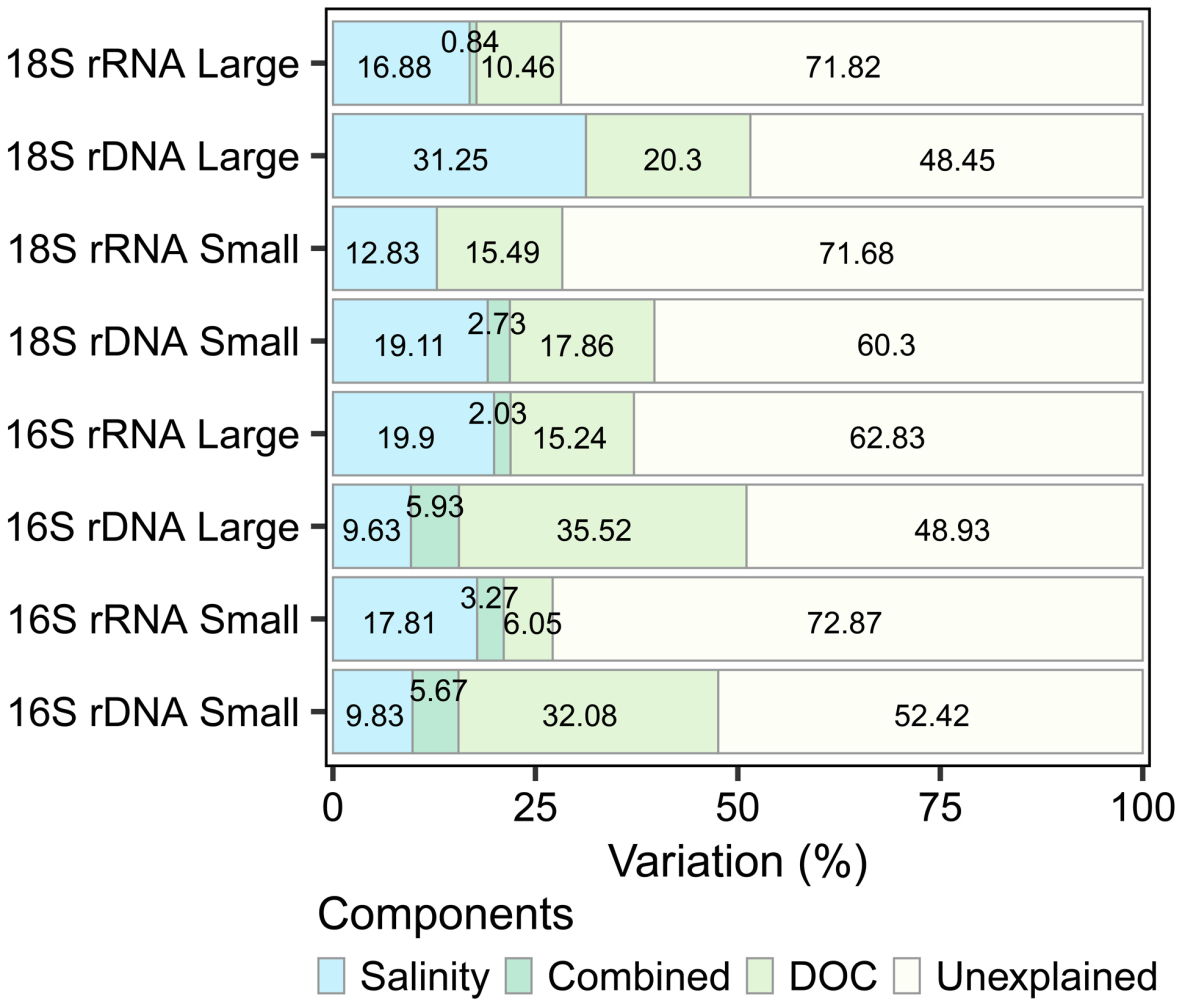
Variation partitioning of the significant environmental drivers (salinity and dissolved organic carbon) for each dataset.

Distance-based redundancy analysis (db-RDA) showed that salinity and DOC were environmental factors that contributed significantly to the variation in microbial eukaryote and bacterioplankton community composition across all sampling sites (*p* < 0.001). The variation explained (adjusted R^2^) was higher for the rDNA and for the large fraction in both the bacterioplankton (rDNA large 51%, small 48%; rRNA large 37%, small 27%) and the microbial eukaryotes (rDNA large 52%, small 40%; rRNA large and small 28%). DOC explained a greater proportion of the variation in bacterioplankton rDNA and the small fraction of microbial eukaryote rRNA, whereas salinity explained more variation in the bacterioplankton rRNA and all other microbial eukaryote samples (Figure 7).

**Figure 8:**
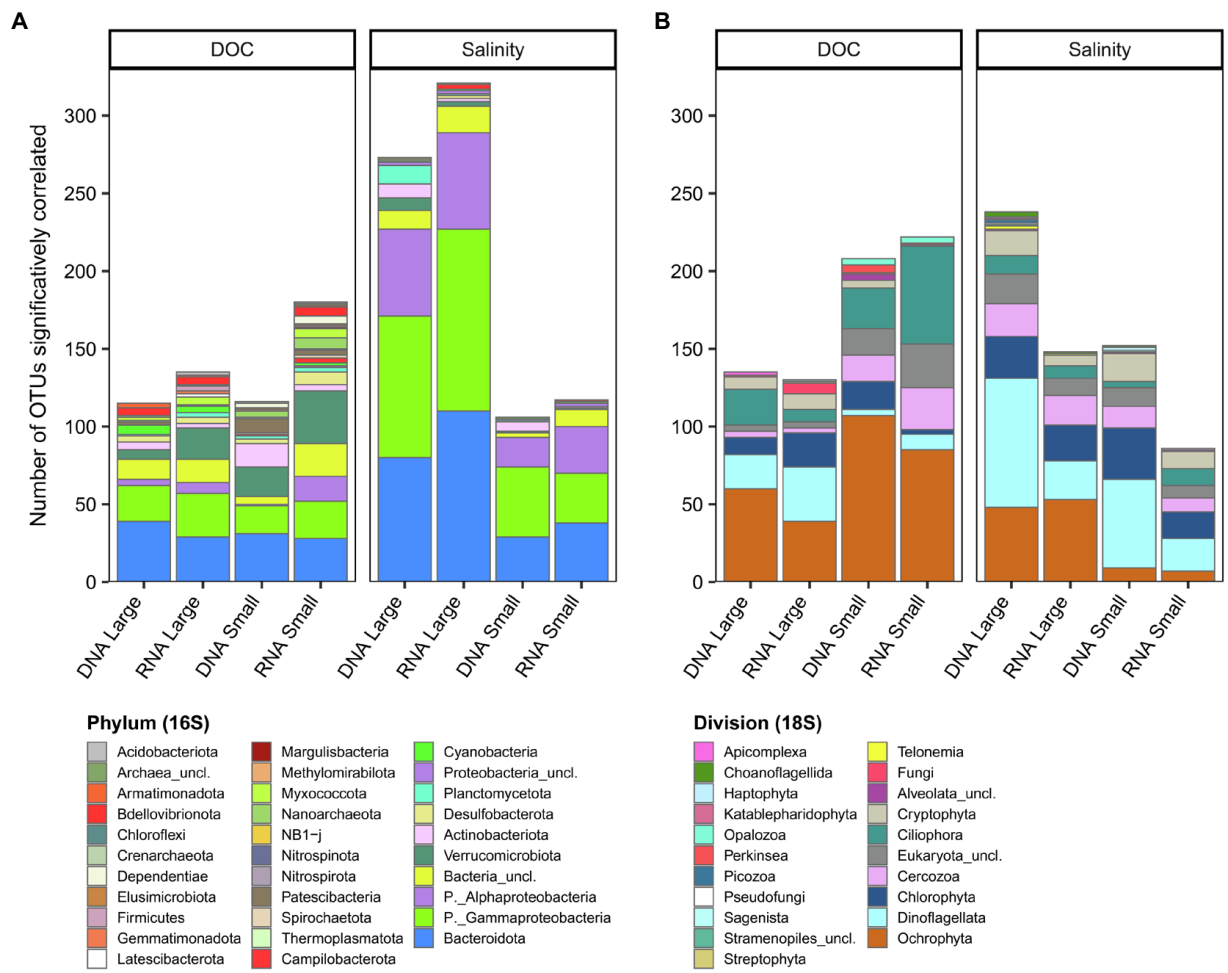
Number of OTUs significantly correlated to DOC or salinity for each rDNA or rRNA fraction for bacterioplankton **(A)** and microbial eukaryotes **(B)**. The OTUs are grouped at the phylum level for bacterioplankton **(A)** and division level for microbial eukaryotes **(B)**.

To identify groups of OTUs (subnetworks) correlating with these potential environmental drivers, we used a weighted correlation network analysis (WGCNA) that was computed separately for bacterioplankton and microbial eukaryotes, and for each fraction and nucleic acid. We then considered OTUs only if they had a significant correlation with DOC or salinity (p_a_ < 0.05) and if they were part of a subnetwork that positively and significantly correlated with DOC or salinity (Figure 8, Supplementary Figures S14–S21). The number of OTUs correlated with salinity for the bacterioplankton large fraction (rDNA, 273; rRNA, 321 OTUs) was more than double that for the small fraction (rDNA, 106; rRNA, 117 OTUs), with a greater number of OTUs principally in the phyla Bacteroidota and Proteobacteria (Gammaproteobacteria and Alphaproteobacteria). The phylum diversity of OTUs correlated to DOC was greater than for salinity (mean 18.5 vs. 10), and was mainly taxa in the Bacteroidota, Proteobacteria (Gammaproteobacteria) and Verrucomicrobiota. More eukaryotic OTUs were correlated with DOC in both the small fraction (rDNA, 208; rRNA, 232) and large fraction (rDNA, 135; rRNA, 130 OTUs), with greater representation of the divisions Ochrophyta and Cercozoa. Dinoflagellata, Ochrophyta and Chlorophyta represented more than half of the eukaryotic OTUs that correlated with salinity.

## Discussion

### Environmental Characteristics

Our sampled environments encompassed a broad range of salinities (0.01 to 14.86), temperatures (11.65 to 19.48°C), TSS (0.96 to 10.89 mg L^−1^), DOC (2.86 to 8.16 mg C L^−1^), TP (<5 to 26.14 μg P L^−1^) and TN (170.30 to 404.48 μg N L^−1^). As hypothesized, permafrost thaw related variables differed among subcatchments, with higher values (except for the TSS) in the SAS and KWK rivers that pass through degraded permafrost landscapes. There was little change over the 150-km reach of GWR in TN, TP and DOC (including CDOM optical variables), suggesting relatively homogenous landscape conditions over much of this section of the catchment, despite some shifts in vegetation and local fire effects (Payette et al., 1989; Bhiry et al., 2011). This relative stability of the GWR environment indicates substantial dilution of the three tributary rivers (KWK, Coats and Denys), which all had higher DOC concentrations than the main stem of the river. There was a downstream increase in TSS along the GWR transect (especially at site G6) that may indicate decreased landscape stability and increased erosion in the lower reaches of the river. Geomorphological analysis of the downstream river channel shows evidence of increased recent landslide activity, possibly linked to increasing extreme rainfall events (Owczarek et al., 2020), and a major landslide occurred 8 km upstream from the river mouth in April 2021 (Nozais et al., 2021). There was a further upshift in TSS between the most downstream freshwater sites (G7, G8) and the plume in coastal Hudson Bay, which may be the result of tidal resuspension of shallow sediments (Ingram, 1981), as well as flocculation of particles induced by the mixing with saline waters (Mosley and Liss, 2020).

The two small rivers (SAS and KWK) differed from the other waters in their physicochemical characteristics, which may in part be due to their lesser size (Wologo et al., 2021), but most likely reflected the influence of severely degrading permafrost in their catchments (Arlen-Pouliot and Bhiry, 2005; Bouchard et al., 2014). These rivers had the highest nutrient, DOC and CDOM (*a*_320_) values, and optical analysis of the CDOM (S_289_, SUVA_254_, S_R_) showed that this carbon was dominated by aromatic compounds of higher molecular weight, indicative of terrestrial rather than autochthonous sources. This allochthonous dominance of DOC is consistent with the allochthonous CDOM previously observed in thermokarst lakes of the SAS and KWK river valleys and attributed to permafrost degradation (Wauthy et al., 2018). The SAS valley contains permafrost peatlands, and peatlands in general have higher DOC export and concentrations than mineral soils (Dillon and Molot, 1997). Radiocarbon analysis of DOC in the SAS River during winter has indicated much older carbon than in nearby non-peatland rivers, and thermokarst lakes in the KWK River watershed (Gonzalez Moguel et al., 2021), suggesting the mobilization of old frozen peat deposits. In a study of streams flowing through discontinuous permafrost, higher concentrations of DON, as well as higher DOC and lower DOC quality, were associated with a greater extent of permafrost in the watershed (Balcarczyk et al., 2009). The underlying permafrost at KWK and SAS is composed of nutrient-enriched marine clays (Table 2 in Deshpande et al., 2016 for a SAS palsa), and thawing of these soils may have contributed to the higher TN and TP values observed in the KWK and SAS rivers.

### Pigment Composition

A wide range of accessory pigments occurred in all samples, indicating that the phototrophs at all sites had major contributions of taxa from diverse phyla, including cyanobacteria (canthaxanthin), dinoflagellates (peridinin), chlorophytes (lutein), prasinophytes (MgDVP), ochrophytes (fucoxanthin) and cryptophytes (crocoxanthin and alloxanthin). This diversity is consistent with HPLC pigment studies in the lakes of the region, including thaw lakes in the KWK and SAS valleys (Przytulska et al., 2016). The plume itself had a distinctive set of pigment signatures that indicated an abundance of cryptophytes and peridinin-containing dinoflagellates. This is in accord with our molecular analyses in which we detected a greater abundance of dinoflagellates in the plume, particularly the peridinin-rich *Heterocapsa pygmaea*, and although the relative sequence abundance of cryptophytes was not greater in the plume, the molecular data showed the presence of the kleptoplastidic species *Mesodinium rubrum*, which contains cryptophyte chloroplasts and their associated pigments (Gustafson et al., 2000). The conspicuous absence of canthaxanthin in the plume at salinities higher than 5.09 suggests the loss of a subset of freshwater cyanobacteria in response to mixing with seawater. This pigment is especially common in filamentous freshwater taxa of cyanobacteria including members of the Nostocales (Przytulska et al., 2016), and such filaments could be differentially lost by grazing or flocculation in the plume. However, cyanobacteria of the family Nostocaceae were detected in the plume in the molecular data, albeit with a decrease in relative abundance, suggesting a limitation of the HPLC analysis. Chlorophyll-*a* concentrations in all samples were within the oligotrophic to mesotrophic range (0.36–3.08 μg chl *a* L^−1^, trophic state index 21.14–40.96; Carlson, 1977). Maximum chlorophyll-*a* concentrations were in the SAS and KWK rivers, consistent with their higher phytoplankton cell concentrations as measured by flow cytometry.

### Core Microbiome

Despite the broad environmental gradients sampled here, a large fraction of the bacterioplankton reads consisted of bacterial OTUs that were ubiquitously distributed across all sites. This core microbiome represented a small percentage of the total richness (198 core OTUs for the small fraction and 322 core OTUs for the large fraction, versus 8,673 total OTUs), implying that a subset of generalist taxa can persist in the chemically diverse environments. These core taxa that were retrieved from the rRNA as well as rDNA indicate the potential for protein synthesis (Blazewicz et al., 2013) across this wide range of conditions. However, there was a decrease in the relative abundance of rRNA OTUs in this core assemblage with increasing salinity, which may indicate a decline in the physiological state of many taxa when they encounter seawater. The combined use of rRNA and rDNA provided a more reliable identification of this core assemblage, as rRNA is considered a more accurate guide to the presence of living cells (Li et al., 2017). Among the core microbiome OTUs, there were some well-known cosmopolitan taxa that are found in a wide range of freshwater environments, including *Polynucleobacter* (Hahn and Hoetzinger, 2019), *Limnohabitans* (Kasalický et al., 2013) and CL500-3 (Andrei et al., 2019), also identified in the core community of the Mississippi River (Henson et al., 2018). In addition to freshwater taxa, terrestrial taxa such as *Geobacter*, *Dinghuibacter*, and *Chthoniobacter*, and sediment taxa such as *Sediminibacterium* were also represented in the core, indicating a microbial contribution from soils and sediment to the river.

The eukaryotic core microbiome consisted of fewer OTUs compared to bacterioplankton and accounted for a small fraction of the total eukaryote reads (57 core OTUs for the small fraction and 48 core OTUs for the large fraction versus 3,383 total OTUs). This is consistent with the view that microbial eukaryotes are less metabolically plastic than bacteria and have a narrower niche breadth (Wu et al., 2018). Species sorting in the GWR plume with rising salinity may have especially restricted the number of taxa in this core subset. Eukaryotic OTUs in this core microbiome included taxa found commonly in freshwater such as the putative parasite *Perkinsea* (Lefèvre et al., 2008; Jobard et al., 2020), many Chrysophyceae such as the mixotrophic genus *Dinobryon*, which produces stomatocysts (Piątek et al., 2020) ensuring their survival in unfavorable conditions, and unclassified members of the freshwater Novel Clade 10 (Cercozoa), which contains biflagellate species that can feed on other flagellates (Bass et al., 2018).

### Comparison of Size Fractions

The bacterial and microbial eukaryote communities showed differences between fractions, similar to aquatic ecosystems elsewhere (e.g., Crump et al., 1999; Savio et al., 2015; Henson et al., 2018; Wang et al., 2020), and many OTUs were significantly more abundant in one of the two fractions. Cyanobacteria in the genera *Synechococcus* and *Cyanobium* often account for a large proportion of the freshwater picophytoplankton (Callieri, 2008), and in the present study two cyanobacterial OTUs, belonging to the Synechococcales order (*Cyanobium PCC-6307* and unclassified Cyanobiaceae) were enriched in the small fraction. Previous work on the GWR has identified phycocyanin-rich cells of this type in concentrations of around 10^4^ cells ml^−1^ and although comparable to other phytoplankton, these cell counts were low relative to the bacterial cell counts in the river of around 10^6^ cells ml^−1^ (Rae and Vincent, 1998), as expected. In the present study, most of the cyanobacteria were associated with the large fraction, in keeping with their tendency to form colonies (e.g., *Snowella*) or to be in filamentous form (e.g., *Aphanizomenon*). Eight Synechococcales OTUs (*Cyanobium PCC-6307*) were also more abundant in the large fraction, indicating their tendency to from aggregates (Jezberová and Komárková, 2007; Huber et al., 2017). Certain well-known soil bacteria such as *Citrifermentans*, *Geothrix* and *Geobacter* were enriched in the larger fraction, suggesting an attached lifestyle. These taxa may also have entered the river along with soil particles washed in from the surrounding catchment.

Eukaryotic taxa that sorted according to differences in cell size included picoeukaryotes such as the chlorophyte *Micromonas*, which occurred in the small fraction. In the study by Jacquemot et al. (2021), this common Arctic Ocean genus (Lovejoy et al., 2007) was abundant throughout coastal Hudson Bay. However, larger taxa were also found in the small fraction, which could relate to cell breakup and transfer of cellular debris through the 3 μm filters, or for some species, may be the result of life cycle stages in the smaller fraction. For example, *Perkinsea* produces zoospores in the size range 2–5 μm (Jobard et al., 2020), which would pass through the larger filter.

There was also substantial overlap of OTUs between size fractions for bacteria (rDNA, 77%; rRNA, 86%). It has been suggested that bacteria can alternate between free-living and particle-associated lifestyles (Grossart, 2010), causing similarity between those two communities (Hollibaugh et al., 2000). However, given that we observed the same high overlap for microbial eukaryotes (rDNA 90%, rRNA 87%), the co-occurrence of OTUs in both fractions could also possibly be explained by filtration artifacts such as filter failure, blockage of the 3-μm filters or cellular break-up (Padilla et al., 2015; Cruaud et al., 2019). It should also be noted that for our comparisons of size fractions, we pooled the entire datasets for each fraction to improve the statistical power. This approach may have masked stronger site-specific differences between fractions, as reported elsewhere. For example, there was a significant difference between size fractions among Amazon River plume samples, but no significant difference among river samples (Doherty et al., 2017). Similarly, in a transect of the Mackenzie River to the Beaufort Sea, a significant difference between fractions was only observed in the open sea samples (Ortega-Retuerta et al., 2013). Additionally, our 30 μm prefiltration may have biased comparison between fractions, as large particles and their associated microbial communities were excluded as well as large microbial aggregates and phytoplankton.

### Diversity Patterns

In the GWR, microbial community structure became less similar as the geographic distance between sampling points increased. This distance–decay relationship is often caused by drift and selection, counteracted by dispersal (Hanson et al., 2012). Since most of the environmental characteristics of the GWR were homogeneous over the 150-km reach sampled, selection probably played a limited role. However, neutral processes such as immigration of taxa from sub-catchment inputs (tributaries, groundwater and overland runoff), combined with high dispersal may have contributed to the modest distance–decay relationship observed. These inputs also likely drove the increase in richness in the free-living bacteria and large microbial eukaryote fraction with distance downstream, rather than growth, competition and replacement processes, given that the total transit time over this reach is relatively short (1 to 3.5 days at flows in the range 0.5–1.5 m s^−1^). This wholesale mixing of different sub-communities from different source locations would be consistent with the concept of longitudinal coalescence in fluvial microbiomes (Mansour et al., 2018). No downstream trends in richness were observed in the particle-associated bacterial fraction, nor in the small eukaryotes, suggesting less heterogeneity in subcatchment inputs for these fractions. Decreases in bacterial diversity have been observed downstream of the headwaters of some systems (Crump et al., 2012; Savio et al., 2015), and such changes could potentially occur at the outflow of the lakes in the headwaters of the GWR, several hundred km upstream of our transect (7 days upstream at 1 m s^−1^).

There was a decrease in richness in the large microbial eukaryote fraction with increasing salinity in the GWR plume. A similar decrease has been reported in the Chesapeake Bay and the Baltic Sea where diversity was higher in both fresh and marine waters and decreased at salinity around 7 to 9, following the Remane curve (Olli et al., 2019). Salinity is a strong filter for dispersal (Lozupone and Knight, 2007) and this observed decrease is likely the result of species sorting of freshwater taxa. Microbial eukaryotes appear to be more subject to species sorting relative to dispersal limitation than bacteria (Wu et al., 2018), which could potentially explain our observed absence of a diversity pattern for bacteria across the plume salinity gradient. Differences in our filtered water volumes could have affected community diversity (Padilla et al., 2015), however we found no correlation between richness and filtered volume in the GWR or plume.

Bacterial richness was higher for the particle-associated fraction than for the free-living fraction at all sites as reported elsewhere and has been attributed to environmental heterogeneity such as redox gradients within the suspended particles (Savio et al., 2015; Payne et al., 2017; Wang et al., 2020). Microbial eukaryote richness was higher for the small fraction in the SAS and KWK rivers suggesting that smaller eukaryotes could potentially be favored in these CDOM-rich waters. All of these analyses are subject to the usual limitations of primer mismatches and amplicon biases (e.g., Burki et al., 2021; Vaulot et al., 2021).

### Salinity Effects

The plume community consisted of OTUs originating from the river, but also specific to the plume, with changes in community composition at salinities as low as 1.16, and a marked change between low (1.16 to 5.09) and moderate (7.58 to 10.07) salinity samples for some eukaryotic taxa. Since we did not sample offshore marine waters in Hudson Bay, we cannot differentiate brackish from marine taxa. However, the earlier study by Jacquemot et al. (2021) in the GWR estuary reported that the eukaryotic microbial community consisted of marine, freshwater, and specialized estuarine taxa. This mixing of taxa from different water sources and the establishment of a brackish population has also been observed for bacteria in transition zones elsewhere, such as in Parker River estuary and Plum Island Sound (Crump et al., 2004) and in the Baltic Sea (Herlemann et al., 2011).

Microbiome structure was significantly related to salinity, as hypothesized, but with less responsiveness of bacteria relative to eukaryotes. Many bacterial taxa occurred across the full range of salinities, however there was a greater number of bacterial OTUs correlated to salinity in the large fraction, suggesting some differentiation of particle-associated bacterial assemblages with increasing saltwater influence in the plume. Bacterial OTUs correlated with salinity were primarily members of the taxonomic groups Bacteroidota, Gammaproteobacteria and Alphaproteobacteria, which often reported as more abundant in the sea compared to freshwater (Bouvier and del Giorgio, 2002; Herlemann et al., 2011). In the GWR system, members of the Rhodobacteraceae family (Alphaproteobacteria), unclassified taxa and the genus *Plantomarina*, were highly correlated with salinity and were almost exclusively in our plume samples. Rhodobacteraceae sometimes dominate coastal marine particles (Bižić-Ionescu et al., 2015) and are abundant over a wide range of salinities including mesohaline waters (Zhu et al., 2018), a hypersaline coastal lagoon (Ghai et al., 2012), and in biofilms over marine macroalgae (Bengtsson et al., 2011; Dogs et al., 2017).

Bacteroidota in the families Cryomorphaceae and Flavobacteriaceae also correlated with salinity in the GWR system. Comparative genomic studies have revealed that adhesion to particles is a major property of marine Flavobacteria (Fernández-Gomez et al., 2013), and they are often abundant in marine particle-associated communities (DeLong et al., 1993; Ortega-Retuerta et al., 2013). These groups have the ability to degrade high molecular weight materials (Cottrell and Kirchman, 2000; Kirchman, 2002; Buchan et al., 2014) and are present during marine phytoplankton blooms and their decay, where they colonize aggregates and senescent phytoplankton cells (Abell and Bowman, 2005; Teeling et al., 2012). Moreover, Flavobacteria have been identified as indicator species for the plume environment in a Colombia River-plume-estuary study (Fortunato et al., 2013).

In the third bacterial group correlated with salinity, Gammaproteobacteria, we identified the OM43 clade, a methylotroph in the Methylophilaceae family (Giovannoni et al., 2008), and the SAR92 clade (family Porticoccaceae), which possess genes for the degradation of complex polysaccharides (Klindworth et al., 2014). These two clades are common in coastal waters (Rappé et al., 1997; Morris et al., 2006; Stingl et al., 2007), and a culture study has shown that their growth is stimulated by high molecular weight organic matter (Sosa et al., 2015).

Only one archaeal OTU, identified as an unclassified member of the Marine Group II (Thermoplasmatota), correlated with salinity. This group is the most abundant planktonic archaeal group in surface marine waters and likely plays a role in degradation of high molecular weight proteins and fatty acids (Pereira et al., 2019; Rinke et al., 2019; Tully, 2019).

There were strong relationships between eukaryotic OTUs and salinity, especially for Dinoflagellata, Ochrophyta and Chlorophyta. Diatoms (within the Ochrophyta) were among the most important plume taxa in terms of relative abundance in the large fraction, notably the species *Skeletonema marinoi*. The genus *Skeletonema* is present in coastal and brackish waters (Kooistra et al., 2008; Paulino et al., 2018) and the ability of *Skeletonema marinoi* to growth at low salinity is variable among strains and populations (Balzano et al., 2011). There was a major shift in Dinoflagellata in the plume, with high relative abundance of unclassified Gymnodiniaceae at low salinity, and dominance of *Heterocapsa pygmaea* at moderate salinity. *H. pygmaea* is thought to be mixotrophic (Bretherton et al., 2020), and grazing by this species along with other phagotrophic protists may contribute towards the decline of bacterial cells in the plume. Unlike many marine dinoflagellates, *H. pygmaea* is well known to contain peridinin as an important light-harvesting pigment (Carbonera et al., 1999), consistent with our HPLC observation of this pigment in the plume.

At moderate salinity, OTUs affiliated with *Pyramimonas australis* dominated the Chlorophyta in the large fraction, and were also detected in the small fraction. *P. australis* is a marine species first identified in Terra Nova Bay, Antarctica (Moro et al., 2002) and subsequently identified in Baffin Bay in the Arctic (Gérikas Ribeiro et al., 2020). In addition, two marine picochlorophytes - the cosmopolitan *Bathycoccus prasinos* (Vannier et al., 2016) and *Micromonas* clade B3 - had highest relative abundances in the small fraction of the plume samples.

The mixotrophic ciliate *Mesodinium rubrum* also showed a relationship with salinity. This taxon was in highest abundance at salinity 10.07 and has been previously identified as the most abundant OTU in the GWR estuary at salinity 12.5 (Jacquemot et al., 2021). This species captures and retains plastids from the cryptophytes *Teleaulax gracilis* and *Plagioselmis prolonga* (Rial et al., 2015); these two cryptophytes also occurred in our sequences, mainly at moderate salinity in the GWR plume. As noted above, this presence of *Mesodinium* and the two cryptophytes is consistent with the HPLC data, with the peak in cryptophyte pigments crocoxanthin and alloxanthin at moderate salinity. *P. prolonga* has been recently identified as the haploid life stage of *T. amphioxeia* (Altenburger et al., 2020), also an important plastid donor to *M. rubrum* (Hansen et al., 2012). This haploid life stage appears to be more abundant in summer when irradiance, temperature and grazing pressure are high, but dissolved inorganic nitrogen concentrations are low (Altenburger et al., 2020).

### Permafrost-Related Effects

We hypothesized that in addition to salinity control, there would be a significant effect of permafrost related variables on microbiome structure in the GWR system and its associated waters. Although TSS and TN did not explain any of the community variation, there were significant relationships with DOC. In our constrained ordination, we considered DOC as a proxy for the cluster of permafrost-related variables since it correlated with CDOM concentration (*a*_320_) and CDOM quality (S_R_, S_289_, SUVA_254_), as well as with TP and TDN (the latter two variables explaining less variation than DOC). Although we focus on DOC in this discussion, it should be kept in mind that its correlates, notably changes in DOC composition thus bioavailability, would also have some impact on community composition.

Unlike the salinity-dependent OTUs that were largely restricted to the plume, most of the OTUs that correlated with DOC occurred in all waters, but were especially abundant in the KWK and SAS rivers that pass through rapidly degrading permafrost catchments. Increased CDOM would result in decreased light availability, although light may not be a limiting factor in this environment, particularly given the regular exposure of cells to high irradiances by mixing to the surface in these relatively shallow turbulent rivers. The fact that SAS and KWK rivers had the highest phytoplankton cell concentrations, along with the absence of a positive relationship between chlorophyll-*a* per unit cell volume and DOC, also argue against CDOM-induced light limitation. In part, the positive relationship between OTUs and DOC-correlated variables may reflect the increased supply of land-derived nitrogen and phosphorus from permafrost soils, but the primary effect is likely to be *via* the availability of organic carbon as an energy source for microbial heterotrophs. Although this DOC is largely allochthonous and of high molecular weight, breakdown by photochemical processes at the surface of the water column (Cory et al., 2014) may ensure a continuous supply of more labile substrates. Also, some bacterial taxa such as certain Bacteroidetes and Verrucomicrobia can break down larger organic polymers (Kirchman, 2002; Cabello-Yeves et al., 2017).

The eukaryotic taxa correlated with DOC were mostly Ochrophyta, Chlorophyta, Dinoflagellata, Ciliophora and Cercozoa. Phagotrophy, in the form of mixotrophy or obligate heterotrophy, is a common feature among these groups, which would give them an advantage in higher DOC environments, where bacteria use DOC as a substrate. The ochrophytes that correlated with DOC were mainly identified as unclassified Chrysophyceae; this family includes many mixotrophs (Sanders and Porter, 1988) that can be stimulated by DOM enrichment (Daggett et al., 2015). The dinoflagellate OTUs correlated with DOC were mostly unclassified taxa, and also a species affiliated to *Asulcocephalium miricentonis* that was first described from a Japanese pond (Takahashi et al., 2015). *A. miricentonis* is a photosynthetic dinoflagellate containing peridinin, and is within the family Suessiaceae that contains mixotrophic taxa (Stoecker, 1999).

Bacterial OTUs correlated with DOC were more diverse than those correlated with salinity, including many OTUs that occurred in low relative abundance. The latter included methylotrophs and methanotrophic genera such as *Methylomonas*, *Methylotenera*, *Crenothrix*, *Candidatus methylopumilus* and *pLW-20*. Among the bacterial phyla, Gammaproteobacteria was among those with the highest number of OTUs correlated with DOC along with Bacteroidota and Verrucomicrobiota. Gammaproteobacteria are favored by high DOC concentrations and show preferences for terrestrial organic matter of high molecular weight (Amaral et al., 2016). The genus *Polynucleobacter* was part of the core microbiome, however 3 of the 19 OTUs significantly correlated to DOC with a greater relative abundance of these sequences in the small fraction. It has been hypothesized that some *Polynucleobacter* species are favored by humic matter degradation products (Hutalle-Schmelzer et al., 2010). A species affiliated to *Armatimonas* was among the most common OTUs (in relative abundance) in the rDNA large fraction of the SAS and KWK rivers, although it was in low relative abundance in the rRNA, suggesting a slow growth rate. This group (Armatimonadota) has been detected in a groundwater culture enriched with a mixture of sediment organic matter and bacterial cell lysate, and may possess the metabolic potential to utilize recalcitrant organic matter (Wu et al., 2020).

Archaea were in greater proportion in the SAS River. Fourteen OTUs, mostly belonging to the order Woesearchaeales and identified as unclassified or as the family SCGC AAA011-D5, were correlated to DOC. Woesearchaeota are present in a variety of environments including freshwater lakes (Ortiz-Alvarez and Casamayor, 2016), permafrost (Shcherbakova et al., 2016), wetlands (Narrowe et al., 2017), and sediments (Liu et al., 2021). They have been reported as the dominant archaeal group in Swedish boreal lakes with higher DOM aromaticity (Juottonen et al., 2020), and in Greenland ponds and lakes characterized by higher organic and inorganic carbon and total nitrogen concentrations (Bomberg et al., 2019). Their small genome and limited metabolic capacities suggest a symbiotic or parasitic lifestyle (Castelle et al., 2015, 2021). In anoxic environments, they have a potential role in nitrogen and sulfur cycling, and a syntrophic relationship with methanogenic archaea has been suggested based on their high co-occurrence (Liu et al., 2018, 2021).

## Conclusion

Numerous rivers drain the vast subarctic landscape, and our study of the Great Whale River and associated flowing waters draws attention to their importance as biological habitats for species-rich microbiomes. Our results show the presence of a core microbiome with less commonality among sites in the eukaryotes than in the prokaryotes. The high diversity of taxa that were more site-specific and of much lower relative abundance relative to this core assemblage suggests that these rarer, more specialized taxa may be potentially more vulnerable to ongoing changes in the catchment and river.

Our observations revealed differences between size-fractions in microbial community structure, and trends in richness along the GWR and its plume that differed among microbial groups, implying taxon-specific responses to the environment. The results highlight the distinctive nature of the coastal microbiome, with specific photosynthetic pigment characteristics and a strong influence of freshwater taxa as well as brackish water and fully marine species. Additionally, these results indicate the effects of degrading permafrost, with DOC explaining part of the variation in microbiome structure, and imply that mixotrophic and methanotrophic species may be favored by ongoing landscape thawing and erosion.

We analyzed the GWR microbiome in late summer, the time of maximum temperatures and high biological activity, and before the onset of fall cooling and freeze-up. Subarctic thaw lakes show marked changes in microbiome composition between summer and winter seasons (Vigneron et al., 2019), and the microbiome structure of Arctic rivers also changes markedly through the year (Crump et al., 2009). There is therefore a need to extend these summer observations of a subarctic river and its coastal plume to other seasons, including the winter period of prolonged ice-cover and the spring conditions of ice break-up and peak discharge.

## Data Availability Statement

The molecular datasets generated in this study are available in the NCBI online repository (https://www.ncbi.nlm.nih.gov/; accession number PRJNA744875) and the environmental and HPLC data are deposited in the northern environmental data repository Nordicana D (http://www.cen.ulaval.ca/nordicanad/en_index.aspx; doi: 10.5885/45741CE-38138EC6C8E849AD and doi: 10.5885/45660CE-8B92339884C146D0).

## Author Contributions

MB formulated the research and sampling design with input from WV. MB, WV, and AM conducted the field sampling. WV obtained the funding and logistic support. CL provided infrastructure and advised on molecular approaches. MB analyzed the data. Writing of the manuscript was led by MB with contributions from WV and input from all authors.

## Funding

This research was supported by the Sentinel North program of Université Laval, funded by the Canada First Research Excellence Fund (CFREF). Additional funding and support were provided by the Natural Sciences and Engineering Research Council of Canada (NSERC), the Canada Research Chair program, the Canada Network of Excellence ArcticNet, and the Centre for Northern Studies (CEN).

## Conflict of Interest

The authors declare that the research was conducted in the absence of any commercial or financial relationships that could be construed as a potential conflict of interest.

## Publisher’s Note

All claims expressed in this article are solely those of the authors and do not necessarily represent those of their affiliated organizations, or those of the publisher, the editors and the reviewers. Any product that may be evaluated in this article, or claim that may be made by its manufacturer, is not guaranteed or endorsed by the publisher.
