# Supplementary material for: Size-Fractionated Microbiome Structure in Subarctic Rivers and a Coastal Plume Across DOC and Salinity Gradients
Source: Front Microbiol. 2022 Jan 3;12:760282. doi: 10.3389/fmicb.2021.760282 (PMC8762315; doi:10.3389/fmicb.2021.760282)
Supplement: Supplementary file 1 [file Data_Sheet_1.ZIP › Supplementary_material/GWR_Revised_mat_sup.pdf]

Supplementary material

Supplementary Figures

Figure S1. Boxplot showing chemical and biological data.

Figure S2. Spearman correlation matrix of environmental variables.

Figure S3. Scatterplot showing chemical and biological data in relation to salinity.

Figure S4. CDOM concentration (a_{320}) and the spectral slope ratio (S_R) as a function of salinity.

Figure S5. Boxplot showing HPLC pigment data.

Figure S6. Scatterplot showing pigment data in relation to salinity.

Figure S7. Boxplot showing observed richness for the large (circle) and small (triangle) fractions.

Figure S8. Archaea reads proportion.

Figure S9. Bacterial community composition.

Figure S10. Cyanobacteria reads proportion.

Figure S11. Microbial eukaryotes community composition.

Figure S12. Distance-decay relationship of the microbial eukaryotes.

Figure S13. Distance-decay relationship of the bacterioplankton.

Figure S14. Reads proportion (%) of OTUs positively correlated to salinity for the small bacterioplankton fraction.

Figure S15. Reads proportion (%) of OTUs positively correlated to salinity for the large bacterioplankton fraction.

Figure S16. Reads proportion (%) of OTUs positively correlated to dissolved organic carbon for the small bacterioplankton fraction.

Figure S17. Reads proportion (%) of OTUs positively correlated to dissolved organic carbon for the large bacterioplankton fraction.

Figure S18. Reads proportion (%) of OTUs positively correlated to salinity for the small microbial eukaryote fraction.

Figure S19. Reads proportion (%) of OTUs positively correlated to salinity for the large microbial eukaryote fraction.

Figure S20. Reads proportion (%) of OTUs positively correlated to dissolved organic carbon for the small microbial eukaryote fraction.

Figure S21. Reads proportion (%) of OTUs positively correlated to dissolved organic carbon for the large microbial eukaryote fraction.

Supplementary Tables

Table S1. Amplicon filtration volume (mL).

Table S2. Physicochemical characteristics of each sampling site.

Table S3. Alpha diversity of the bacterioplankton.

Table S4. Alpha diversity of the microbial eukaryotes.

Supplemental Data Files

Data File 1. Fasta file 16S.

Date File 2. Fasta file 18S.

Data File 3. -Table S5. OTU table for the small bacterioplankton fraction core.

-Table S6. OTU table for the large bacterioplankton fraction core.

-Table S7. OTU table for the small microbial eukaryote fraction core.

-Table S8. OTU table for the large microbial eukaryote fraction core.

-OTUs Table 16S.

-OTUs Table 18S.

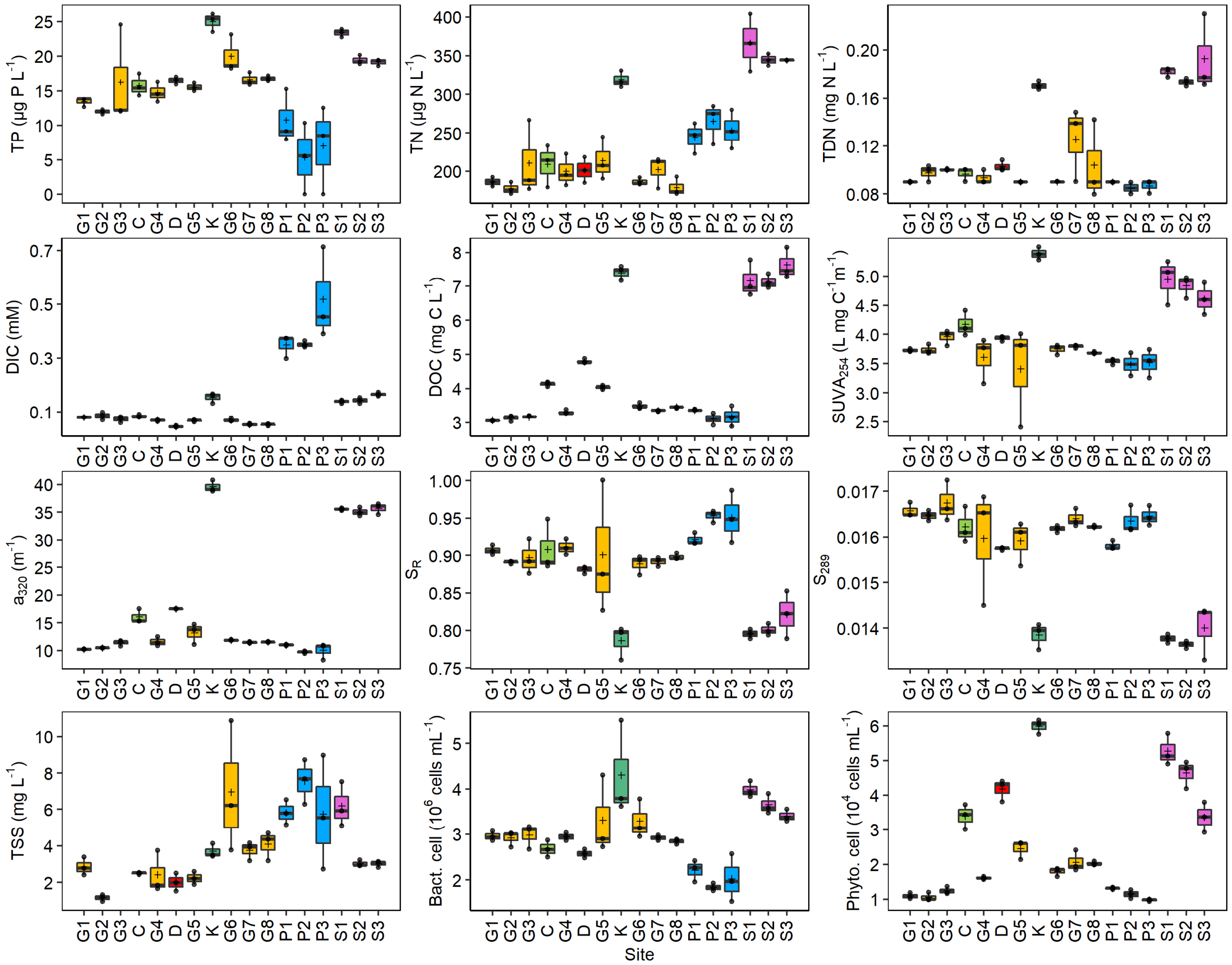

Supplementary Figure S1. Boxplot showing chemical and biological data for the Great Whale River (G), upstream to downstream, including its tributaries (Coats:C, Denys:D, Kwakwatanikapistikw:K) at their confluence, followed by its plume into Hudson Bay (P) and the Sasapimakwananistikw River (S, upstream to downstream). TP: total phosphorus, TN: total nitrogen, TDN: total dissolved nitrogen, DIC: dissolved inorganic carbon, DOC: dissolved organic carbon, $SUVA_{254}$: Specific ultraviolet absorbance at 254 nm, a_{320}: colored dissolved organic carbon absorption coefficient at 320 nm, SR: spectral slope ratio S285/S375, S_{289}: index of autochthonous carbon,TSS: total suspended sediments, n=3 at each site, except TSS G3 n=0, DIC G1 n=1, DOC, $SUVA_{254}$ and TDN P2 n=2. The cross in the boxplot represents the mean.

	Temperature	SR	S289	Bacterial.cell	TDN	SUVA254	a320DOC	TP	a320	DOC	Phytoplankton.cell	Chlorophyll.a	TSS	pH	TN	DIC	Salinity	Conductivity
Temperature	1	0.07	0.32	-0.01	-0.22	-0.21	-0.23	-0.16	-0.25	-0.31	-0.17	-0.19	-0.45	-0.49	-0.63	-0.69	-0.74	-0.79
SR	0.07	1	0.63	-0.78	-0.8	-0.84	-0.81	-0.75	-0.82	-0.76	-0.77	-0.77	0.12	-0.12	-0.41	0.06	0.03	0.02
S289	0.32	0.63	1	-0.47	-0.65	-0.54	-0.64	-0.58	-0.76	-0.79	-0.72	-0.75	-0.12	-0.42	-0.66	-0.26	-0.38	-0.45
Bacterial.cell	-0.01	-0.78	-0.47	1	0.6	0.68	0.68	0.75	0.65	0.57	0.57	0.64	-0.05	0.09	0.33	-0.07	-0.13	-0.21
TDN	-0.22	-0.8	-0.65	0.6	1	0.7	0.74	0.66	0.72	0.69	0.71	0.74	-0.11	0.1	0.46	0.06	0.12	0.11
SUVA254	-0.21	-0.84	-0.54	0.68	0.7	1	0.93	0.74	0.84	0.75	0.82	0.82	-0.1	0.1	0.51	0.03	0.01	0.05
a320DOC	-0.23	-0.81	-0.64	0.68	0.74	0.93	1	0.72	0.9	0.76	0.82	0.87	-0.12	0.14	0.58	0.01	0.02	0.07
TP	-0.16	-0.75	-0.58	0.75	0.66	0.74	0.72	1	0.85	0.81	0.81	0.84	0.15	0.23	0.39	-0.14	-0.07	0.02
a320	-0.25	-0.82	-0.76	0.65	0.72	0.84	0.9	0.85	1	0.94	0.93	0.94	-0.08	0.18	0.53	-0.06	0	0.14
DOC	-0.31	-0.76	-0.79	0.57	0.69	0.75	0.76	0.81	0.94	1	0.91	0.9	-0.08	0.24	0.55	0.02	0.1	0.26
Phytoplankton.cell	-0.17	-0.77	-0.72	0.57	0.71	0.82	0.82	0.81	0.93	0.91	1	0.93	-0.05	0.13	0.43	-0.16	-0.06	0.11
Chlorophyll.a	-0.19	-0.77	-0.75	0.64	0.74	0.82	0.87	0.84	0.94	0.9	0.93	1	0	0.23	0.51	-0.07	0	0.12
TSS	-0.45	0.12	-0.12	-0.05	-0.11	-0.1	-0.12	0.15	-0.08	-0.08	-0.05	0	1	0.64	0.3	0.38	0.54	0.55
pH	-0.49	-0.12	-0.42	0.09	0.1	0.1	0.14	0.23	0.18	0.24	0.13	0.23	0.64	1	0.62	0.59	0.72	0.67
TN	-0.63	-0.41	-0.66	0.33	0.46	0.51	0.58	0.39	0.53	0.55	0.43	0.51	0.3	0.62	1	0.64	0.72	0.68
DIC	-0.69	0.06	-0.26	-0.07	0.06	0.03	0.01	-0.14	-0.06	0.02	-0.16	-0.07	0.38	0.59	0.64	1	0.9	0.75
Salinity	-0.74	0.03	-0.38	-0.13	0.12	0.01	0.02	-0.07	0	0.1	-0.06	0	0.54	0.72	0.72	0.9	1	0.91
Conductivity	-0.79	0.02	-0.45	-0.21	0.11	0.05	0.07	0.02	0.14	0.26	0.11	0.12	0.55	0.67	0.68	0.75	0.91	1

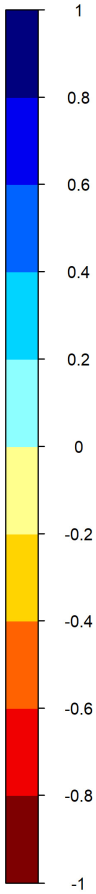

Supplementary Figure S2. Spearman correlation matrix of environmental variables. SR: spectral slope ratio S285/S375, S289: index of autochthonous carbon, TDN: total dissolved nitrogen, SUVA254: Specific ultraviolet absorbance at 254 nm, a320DOC: colored dissolved organic carbon absorbance at 320 nm normalized by dissolved organic carbon concentration, TP: total phosphorus, a320: colored dissolved organic carbon absorbance at 320 nm, DOC: dissolved organic carbon, TSS: total suspended sediments, TN: total nitrogen, DIC: dissolved inorganic carbon.

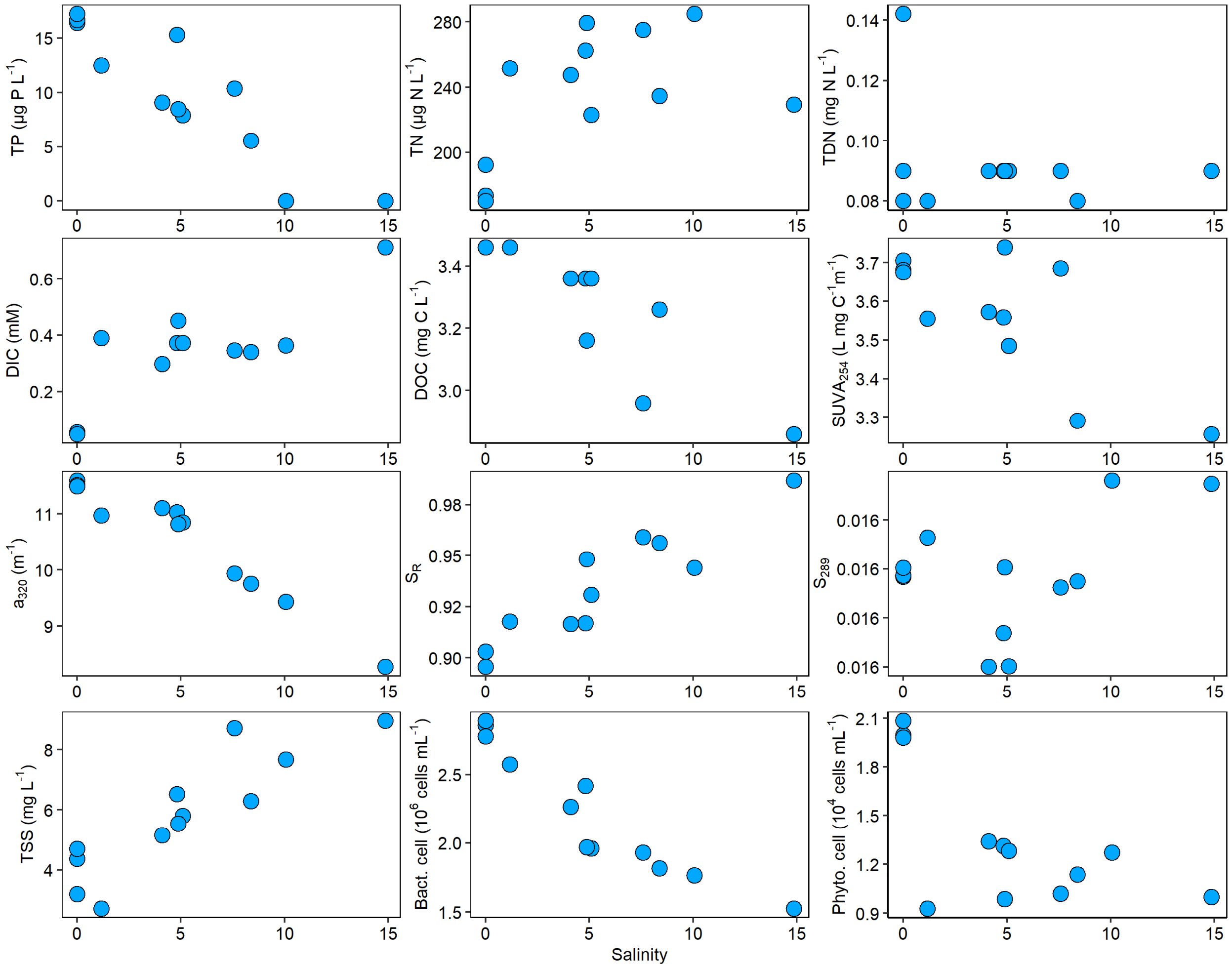

Supplementary Figure S3. Scatterplot showing chemical and biological data in relation to salinity in the plume starting from the Great Whale River mouth. TP: total phosphorus, TN: total nitrogen, TDN: total dissolved nitrogen, DIC: dissolved inorganic carbon, DOC: dissolved organic carbon, $SUVA_{254}$: Specific ultraviolet absorbance at 254 nm, a_{320}: colored dissolved organic carbon absorption coefficient at 320 nm, SR: spectral slope ratio S285/S375, S_{289}: index of autochthonous carbon,TSS: total suspended sediments.

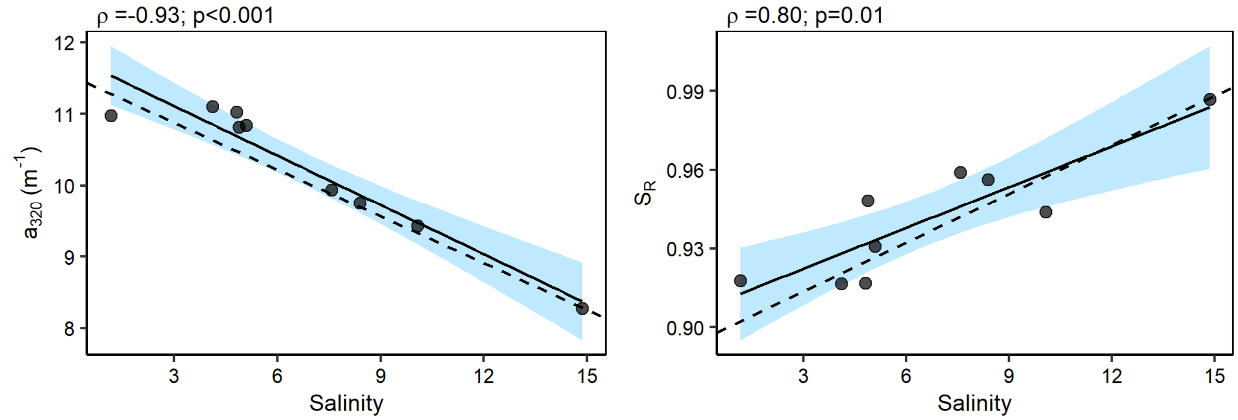

Supplementary Figure S4. CDOM concentration (a_{320}) and the spectral slope ratio (S_R) correlated with salinity in the plume. The dashed line indicates the conservative mixing line, and the blue shade indicates the 95% confidence interval of a linear fit.

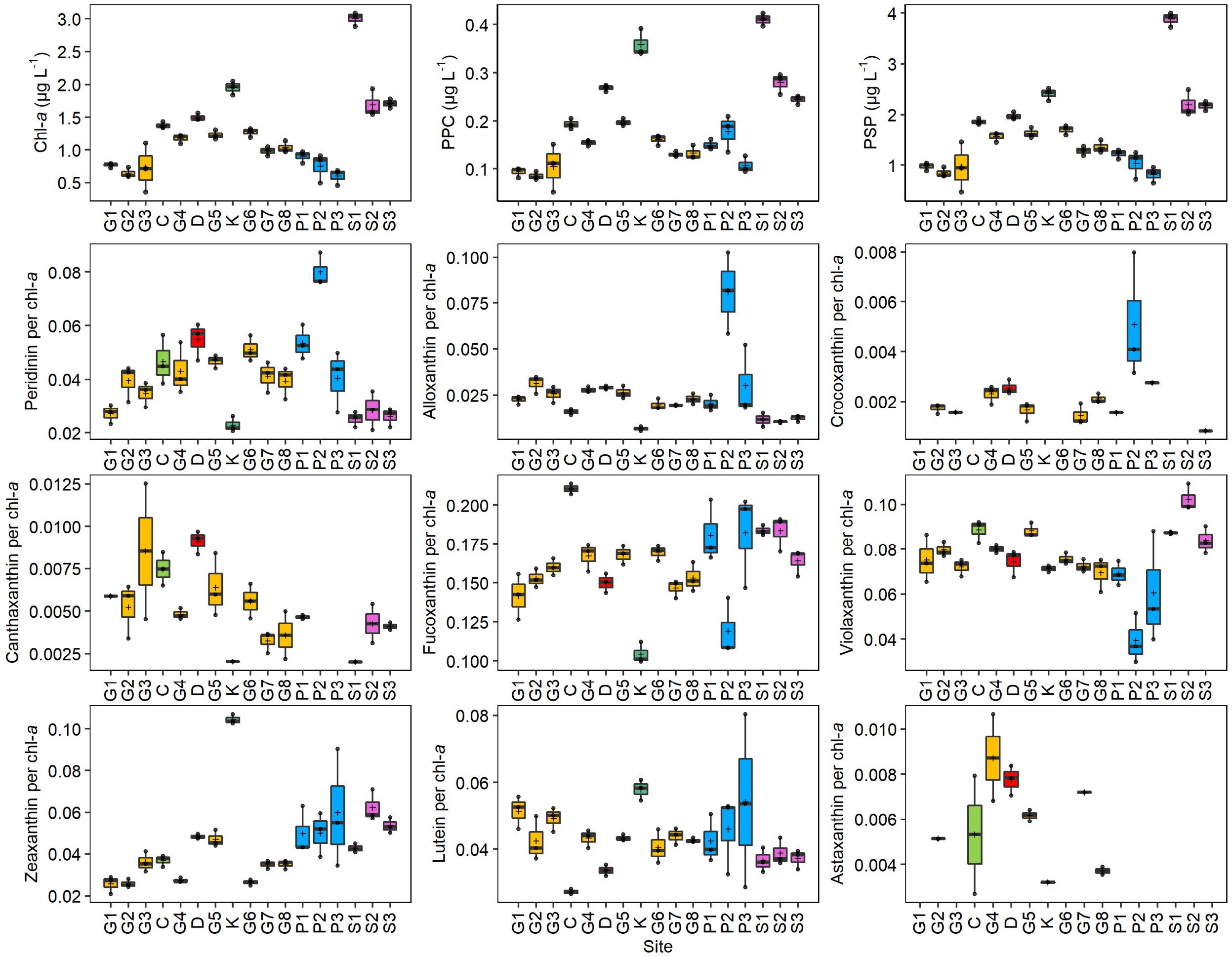

Supplementary Figure S5. Boxplot showing HPLC pigment data for the Great Whale River (G), upstream to downstream, including its tributaries (Coats:C, Denys:D, Kwakwatanikapistikw:K) at their confluence, followed by its plume into Hudson Bay (P) and the Sasapimakwananistikw River (S, upstream to downstream). Chl-*a*: chlorophyll-*a*, PPC: photoprotective carotenoids, PSP: photosynthetic pigments. Except for the chl-*a*, PPC and PSP, pigment concentrations were normalized to chlorophyll-*a* concentration (by weight). The cross in the boxplot represents the mean.

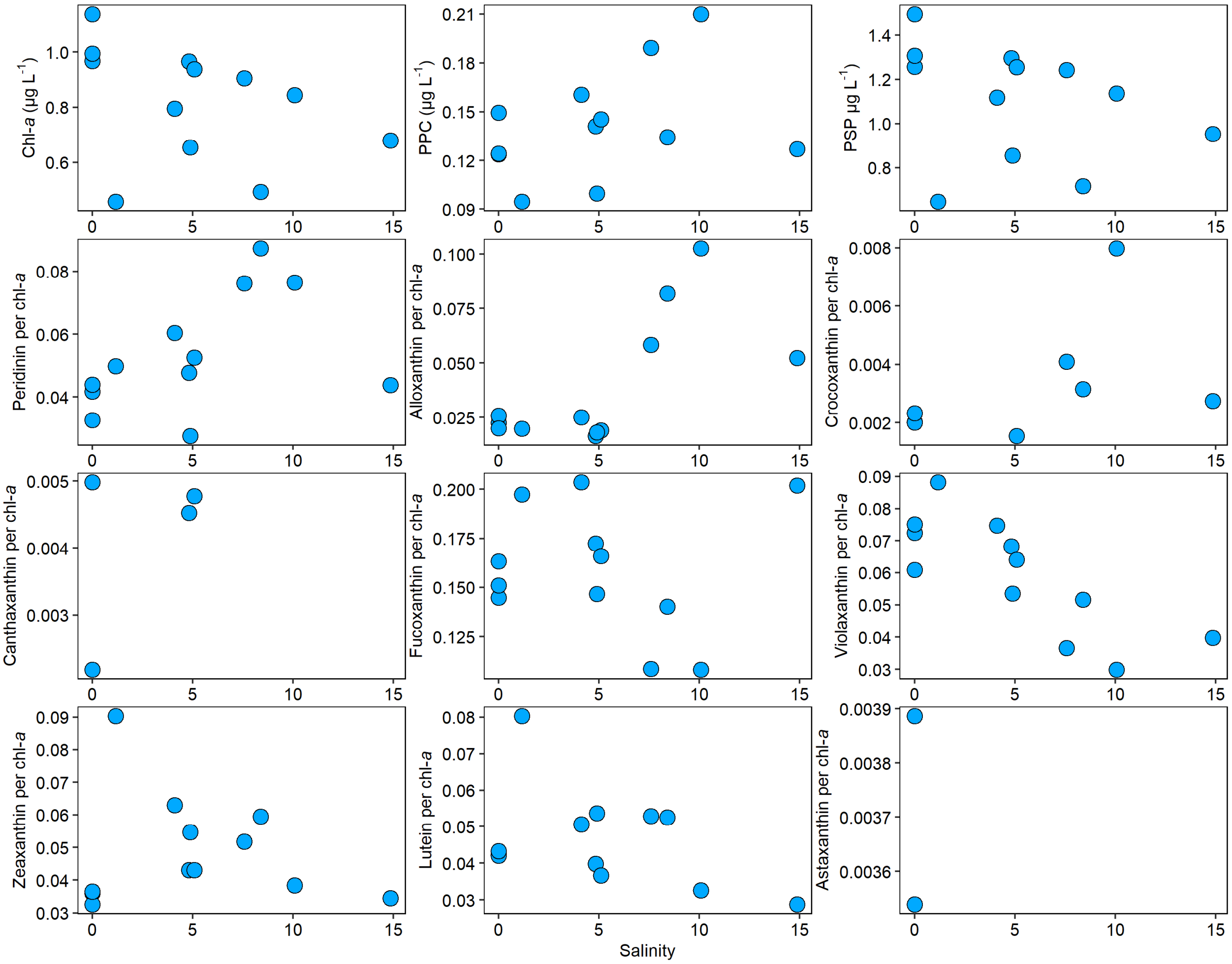

Supplementary Figure S6. Scatterplot showing HPLC pigment data in relation to salinity in the plume starting from the Great Whale River mouth. Chl-*a*: chlorophyll-*a*, PPC: photoprotective carotenoids, PSP: photosynthetic pigments. Except for the chl-*a*, PPC and PSP, pigment concentrations were normalized to chlorophyll-*a* concentration (by weight).

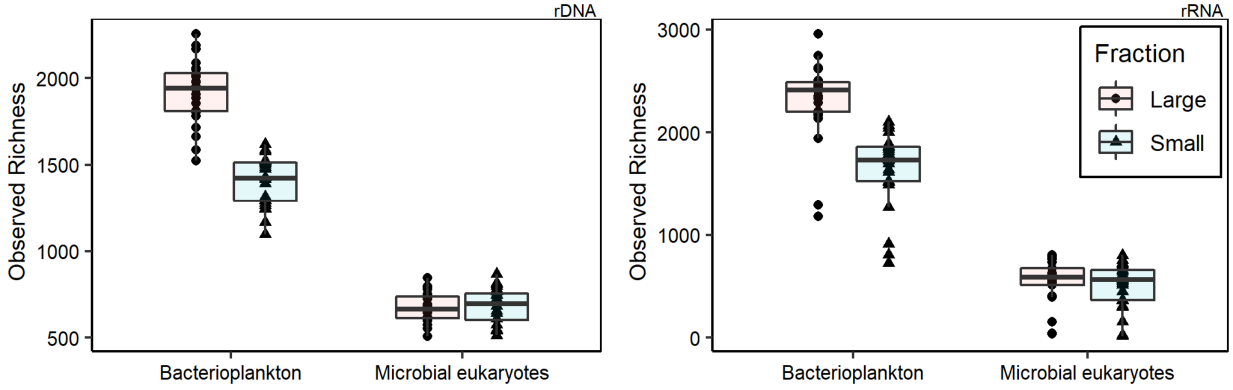

Supplementary Figure S7. Boxplot showing observed richness for the large (circle) and small (triangle) fractions for the bacterioplankton and the microbial eukaryotes in rDNA (left) and rRNA (right).

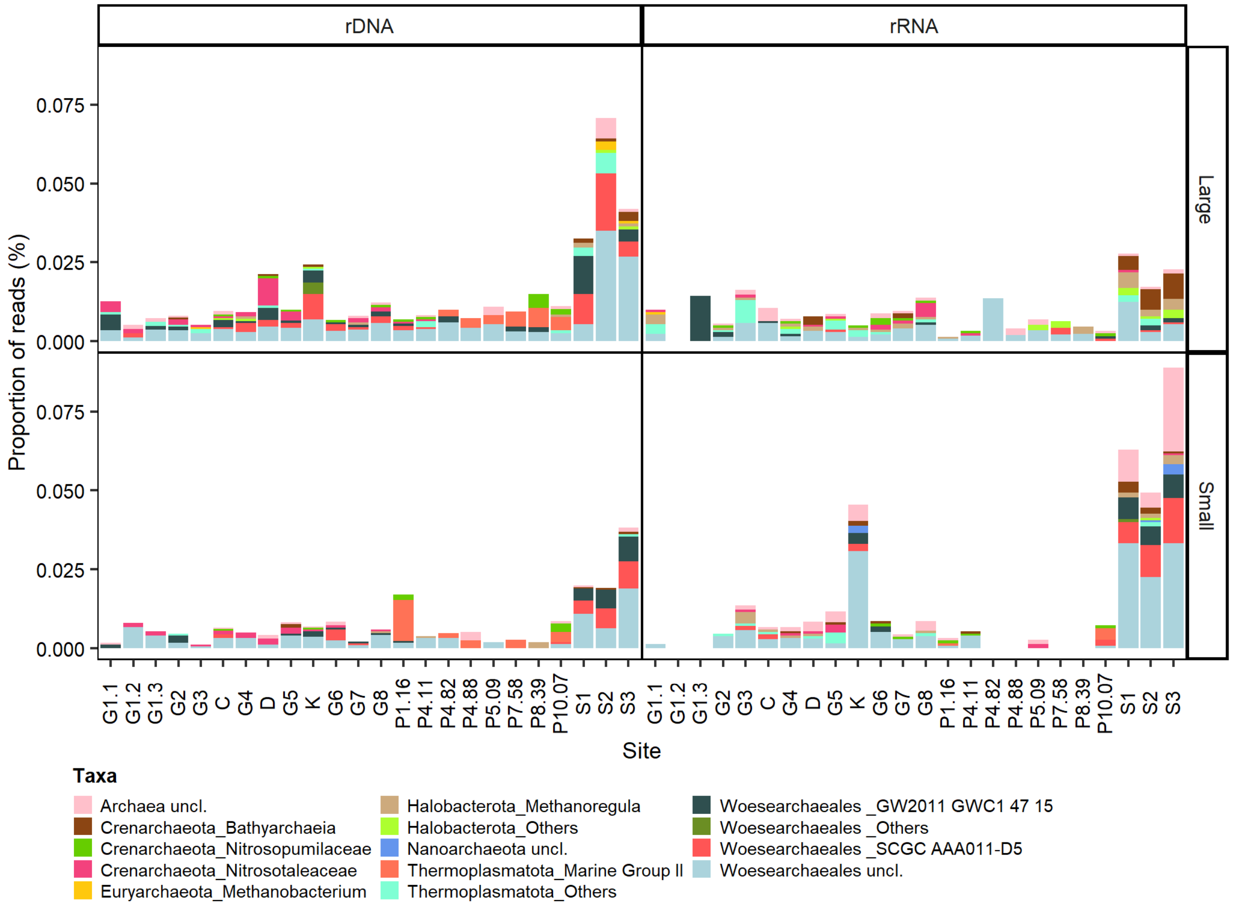

Supplementary Figure S8. Archaea reads proportion (%) for the large (top) and small fraction (bottom) of the rDNA (left) and rRNA (right). GWR upstream to downstream (G), tributaries (C, D, K), plume (by salinity) and SAS River (S, upstream to downstream).

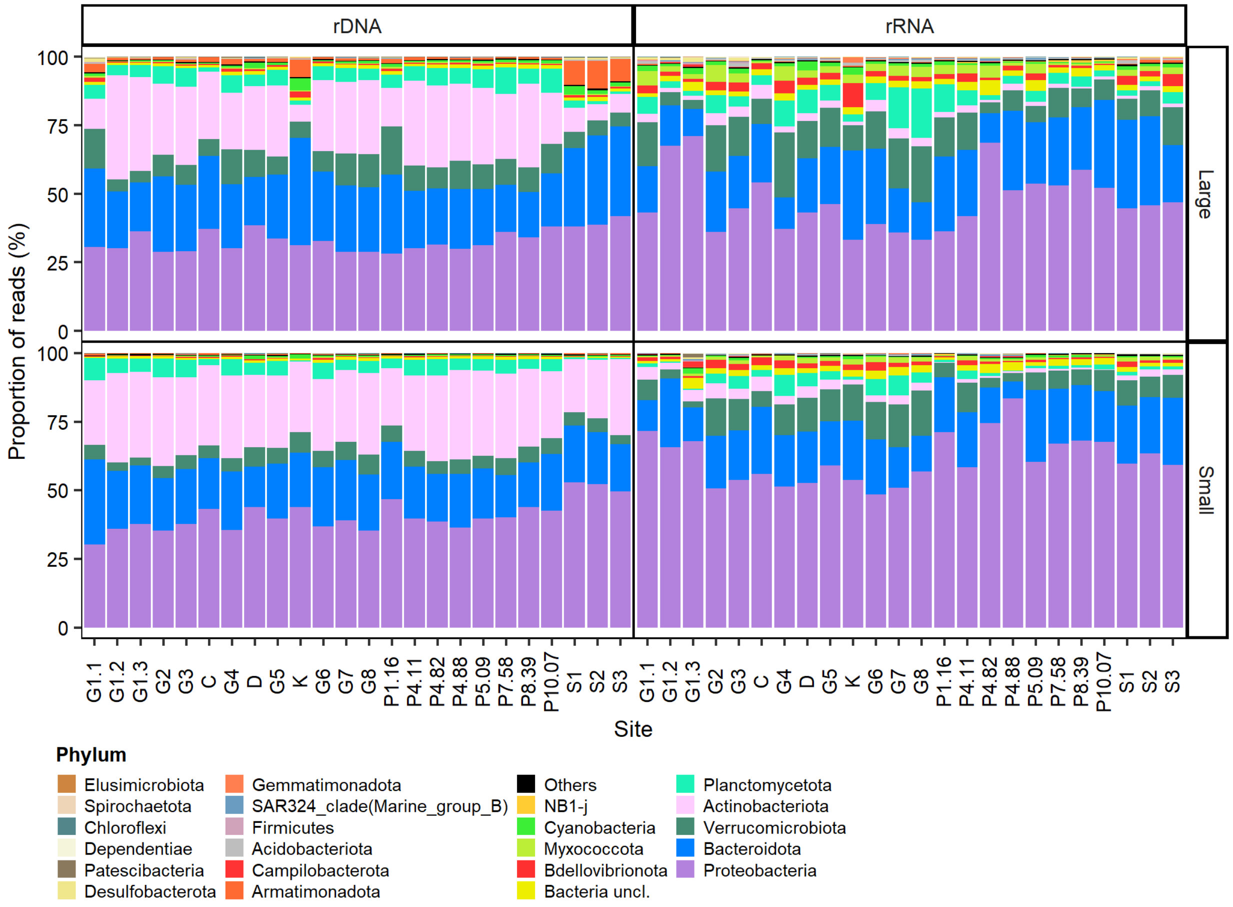

Supplementary Figure S9. Bacterial community composition (reads proportion %) at phylum level for the large (top) and small fraction (bottom) of the rDNA (left) and rRNA (right). GWR upstream to downstream (G), tributaries (C, D, K), plume (P, by salinity) and SAS River (S, upstream to downstream). Others correspond to phylum with proportion smaller than 0.1%.

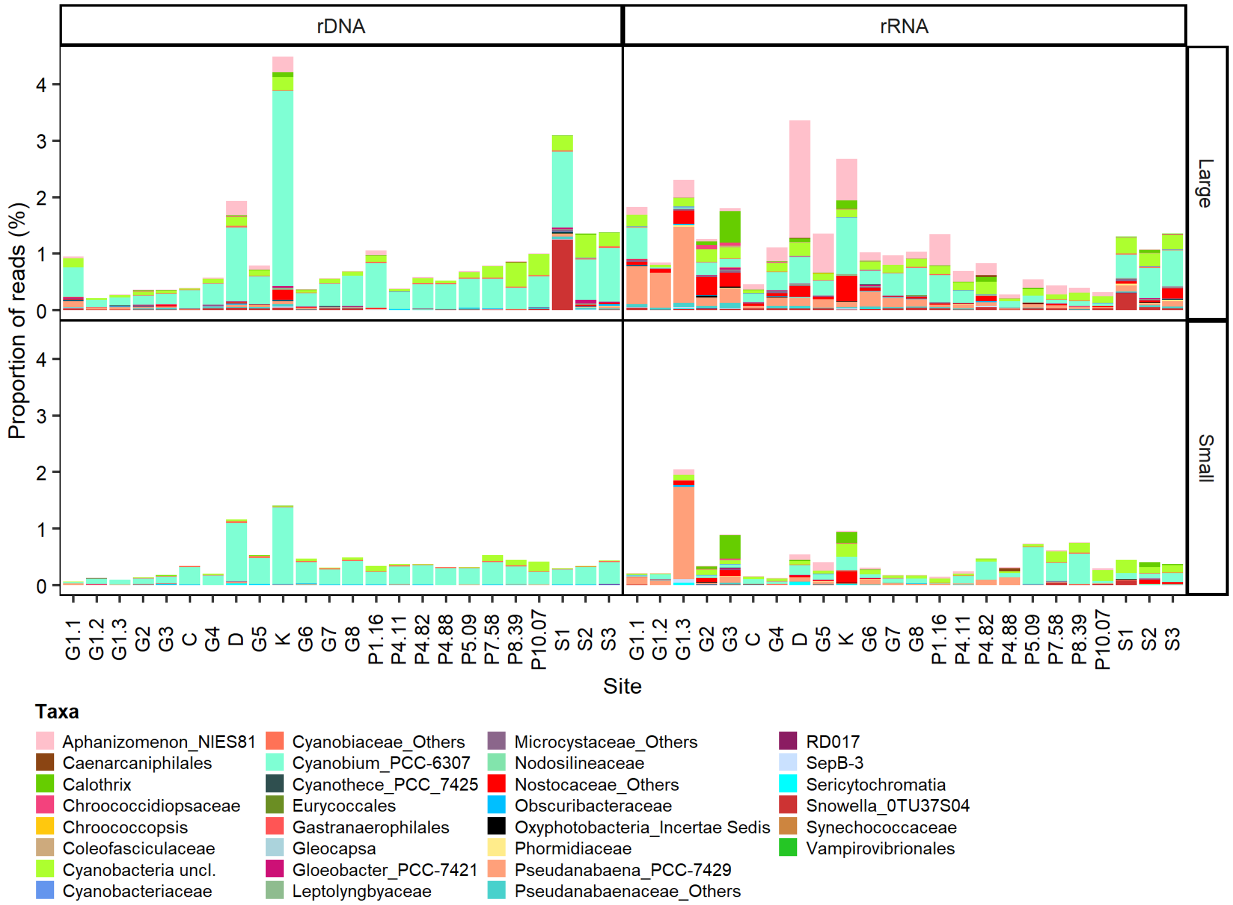

Supplementary Figure S10. Cyanobacteria reads proportion (%) for the large (top) and small fraction (bottom) of the rDNA (left) and rRNA (right). GWR upstream to downstream (G), tributaries (C, D, K), plume (by salinity) and SAS River (S, upstream to downstream).

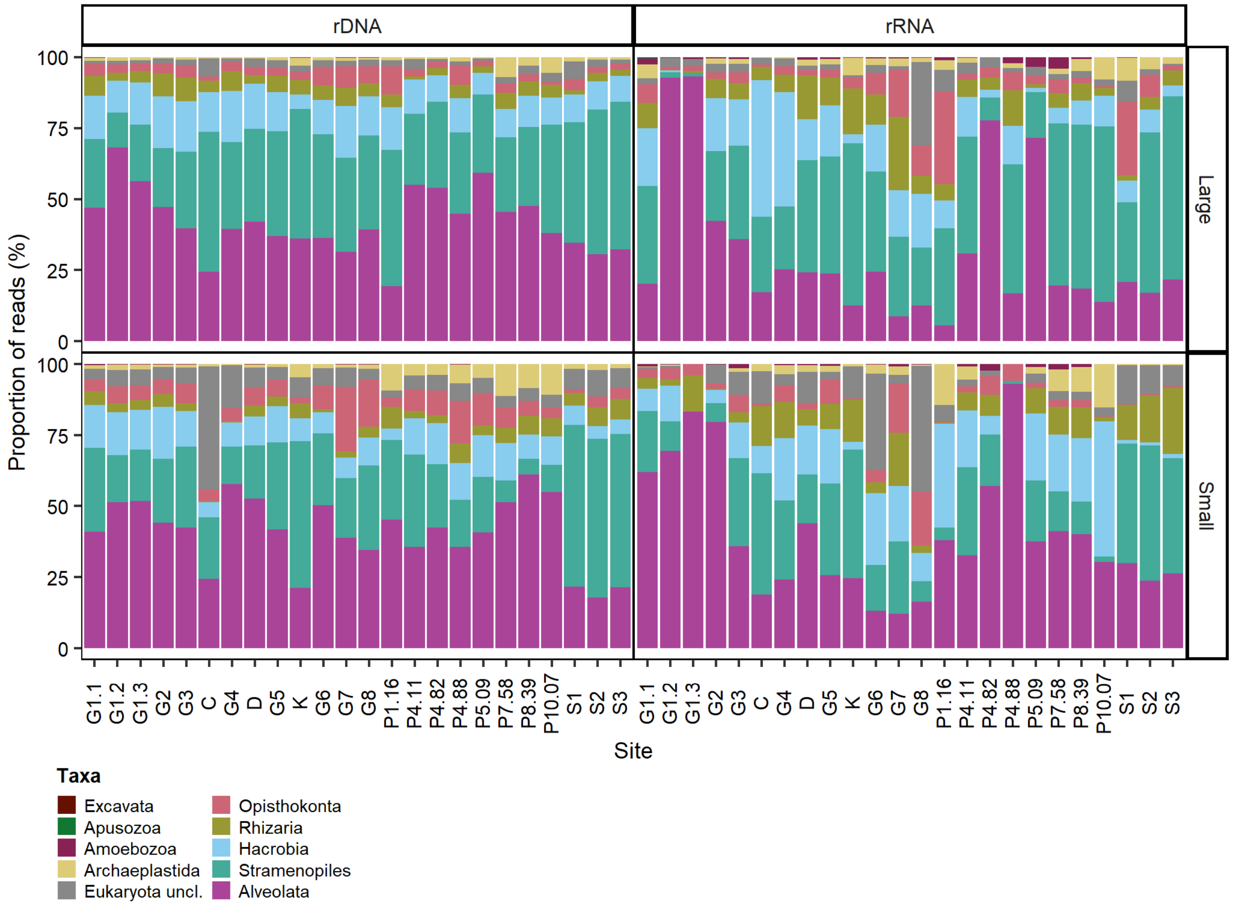

Supplementary Figure S11. Microbial eukaryotes community composition (reads proportion %) for the large (top) and small fraction (bottom) of the rDNA (left) and rRNA (right). GWR upstream to downstream (G), tributaries (C, D, K), plume (by salinity) and SAS River (S, upstream to downstream).

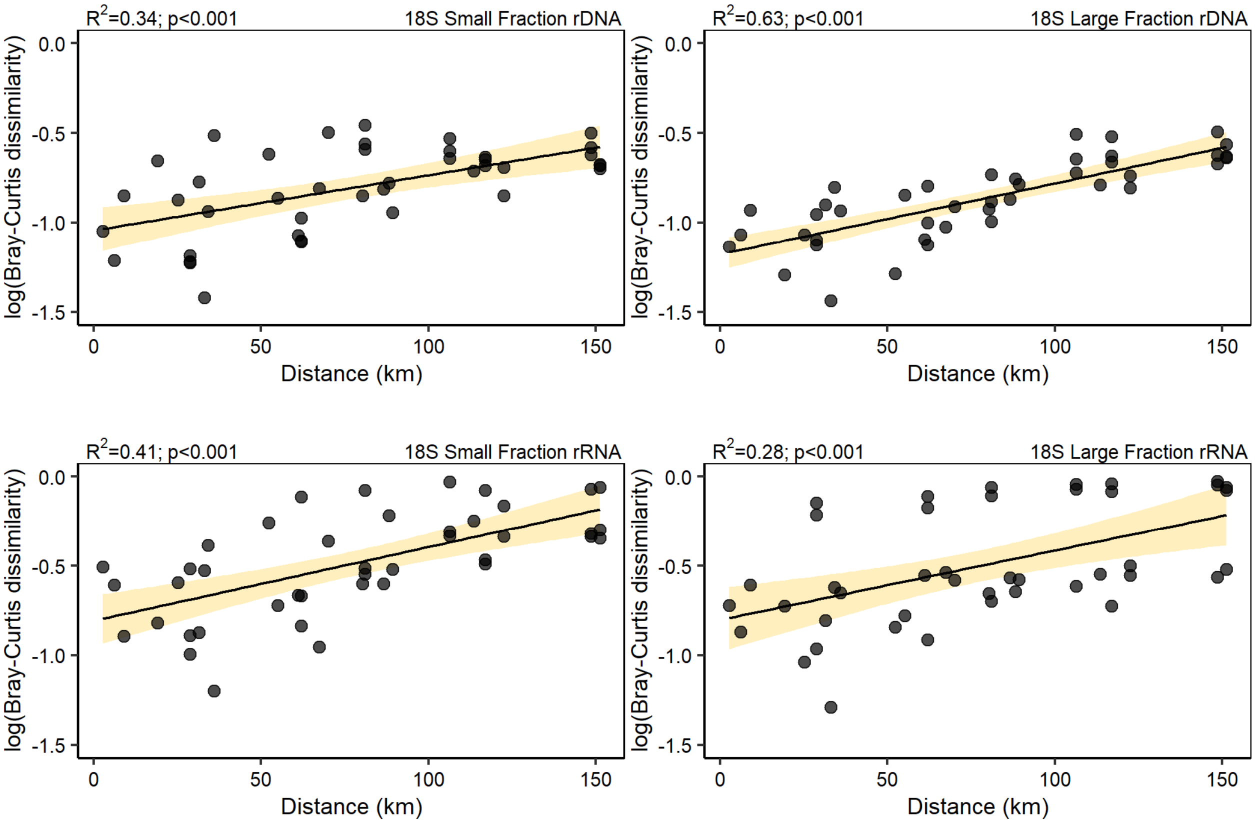

Supplementary Figure S12. Distance-decay relationship of the microbial eukaryotes community for the rDNA (top), rRNA(bottom), small fraction (left) and large fraction (right).

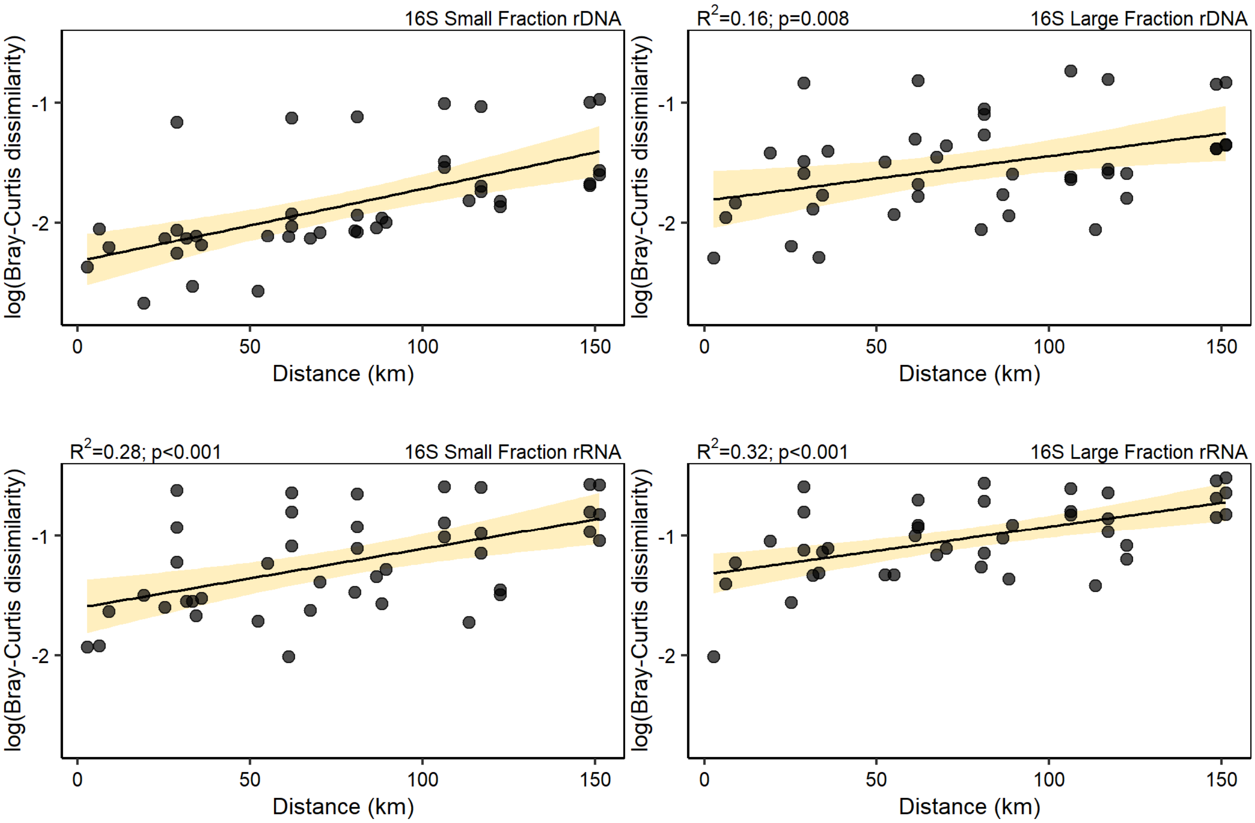

Supplementary Figure S13. Distance-decay relationship of the bacterioplankton community for the rDNA (top), rRNA(bottom), small fraction (left) and large fraction (right).

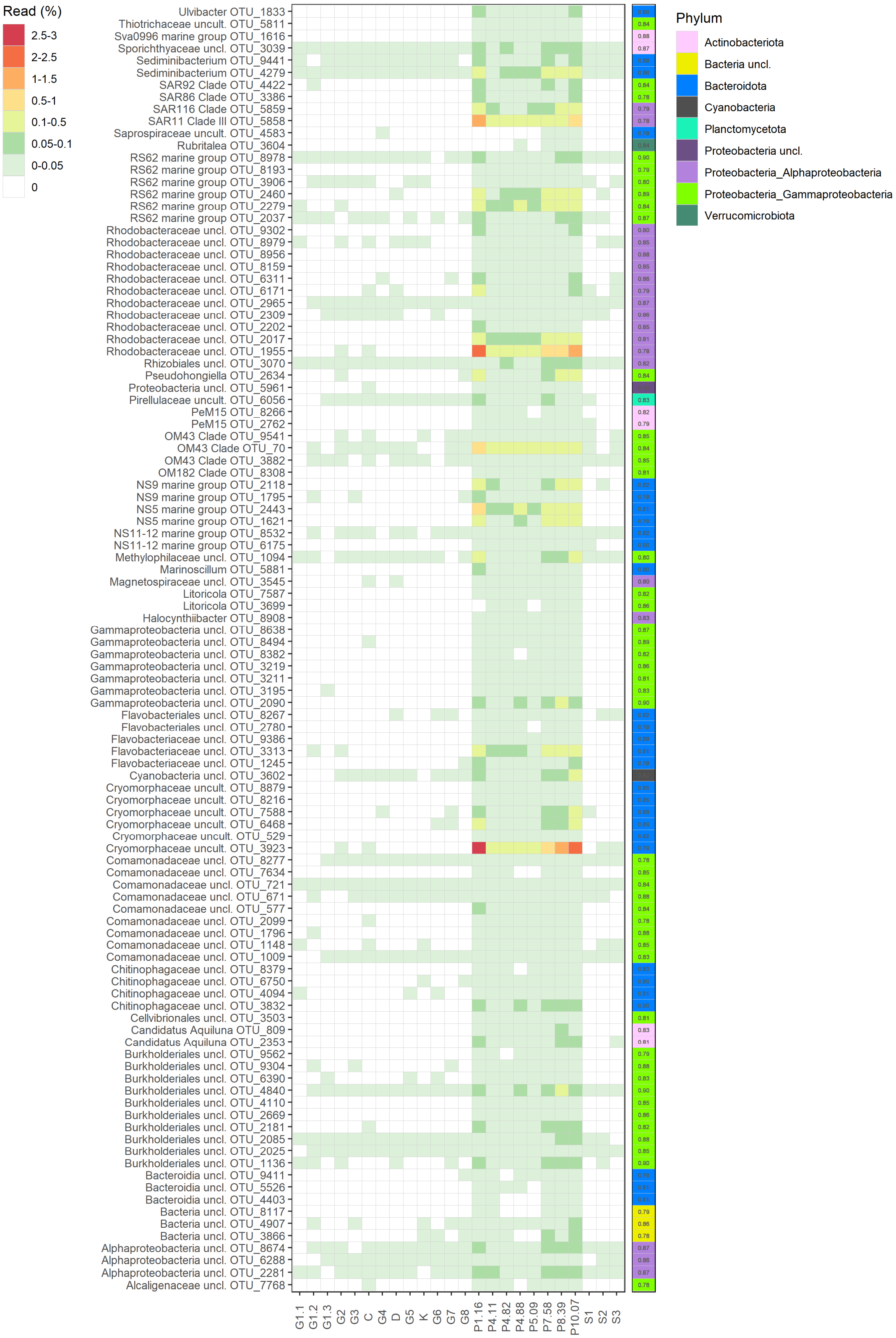

Supplementary Figure S14. Reads proportion (%) of OTUs positively correlated to salinity for the small bacterioplankton fraction in rDNA. The color in the right column indicates phylum and the number correspond to Pearson correlation coefficient.

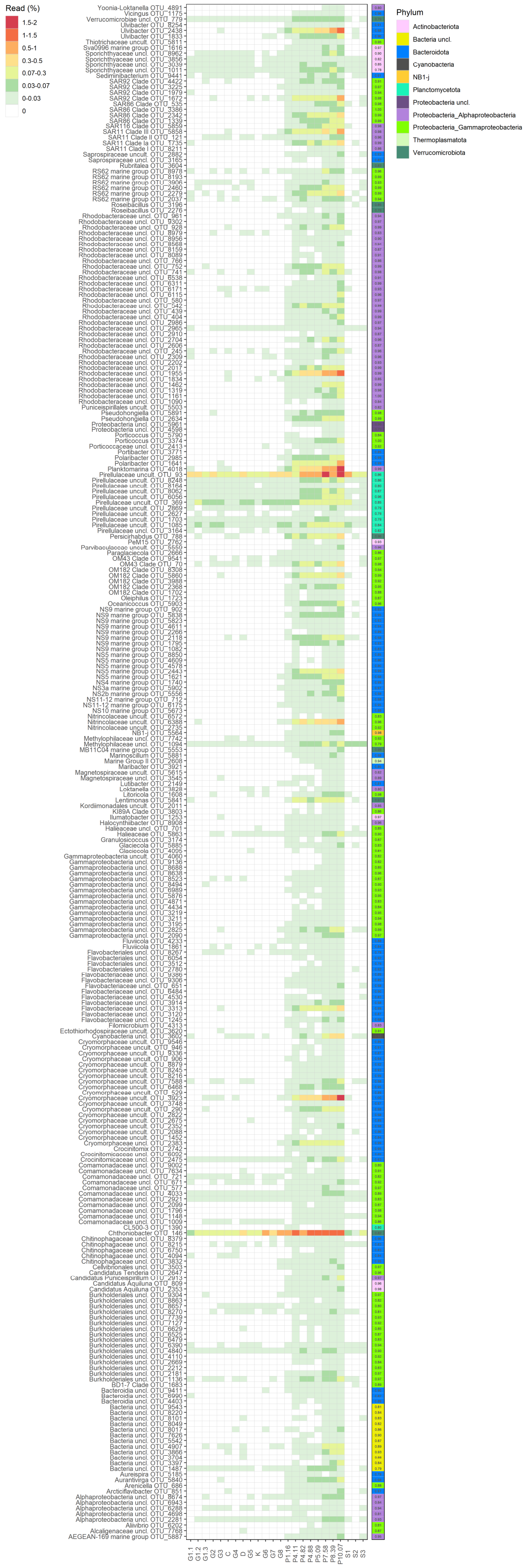

Supplementary Figure S15. Reads proportion (%) of OTUs positively correlated to salinity for the large bacterioplankton fraction in rDNA. The color in the right column indicates phylum and the number correspond to Pearson correlation coefficient.

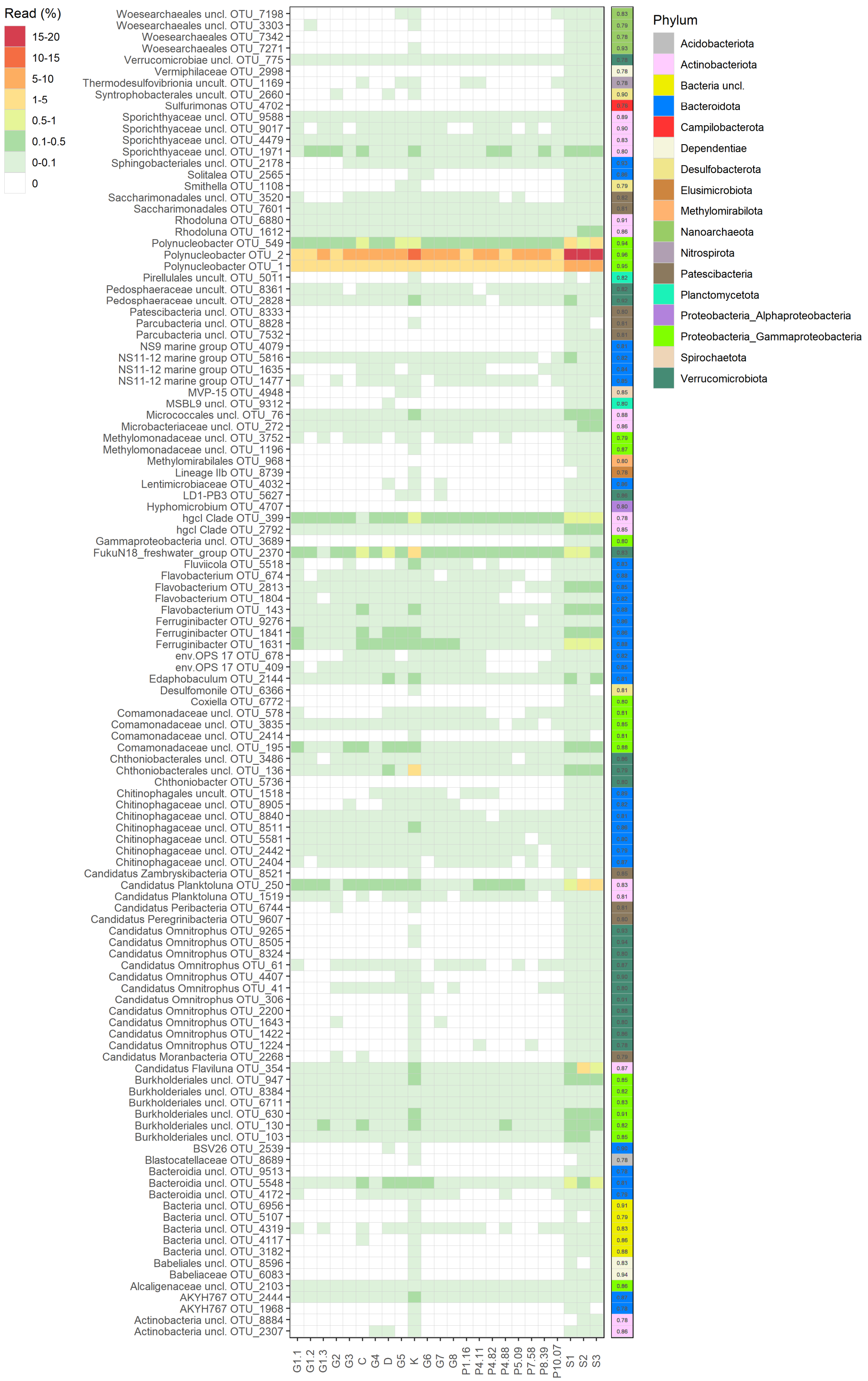

Supplementary Figure S16. Reads proportion (%) of OTUs positively correlated to dissolved organic carbon for the small bacterioplankton fraction in rDNA. The color in the right column indicates phylum and the number correspond to Pearson correlation coefficient.

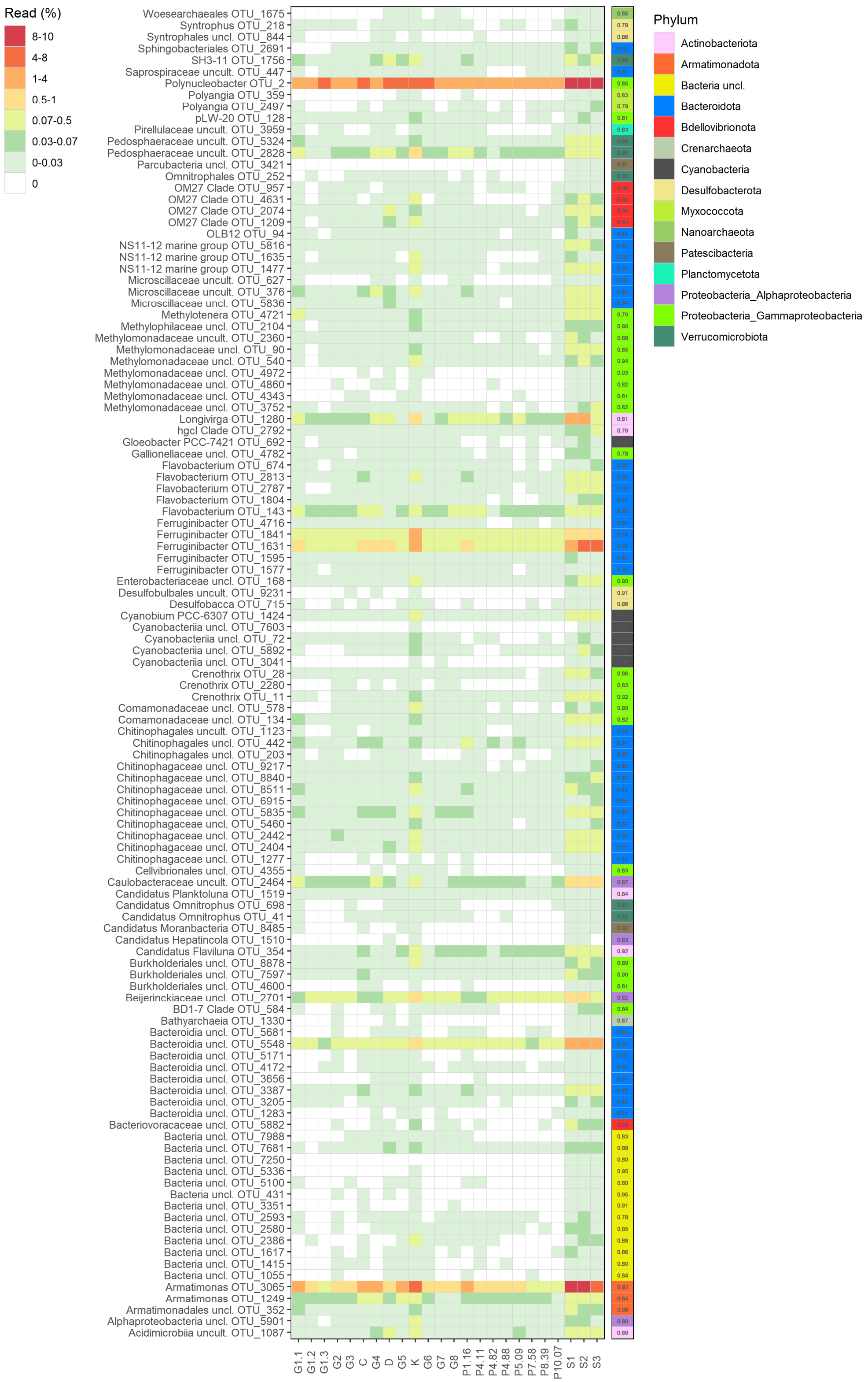

Supplementary Figure S17. Reads proportion (%) of OTUs positively correlated to dissolved organic carbon for the large bacterioplankton fraction in rDNA. The color in the right column indicates phylum and the number correspond to Pearson correlation coefficient.

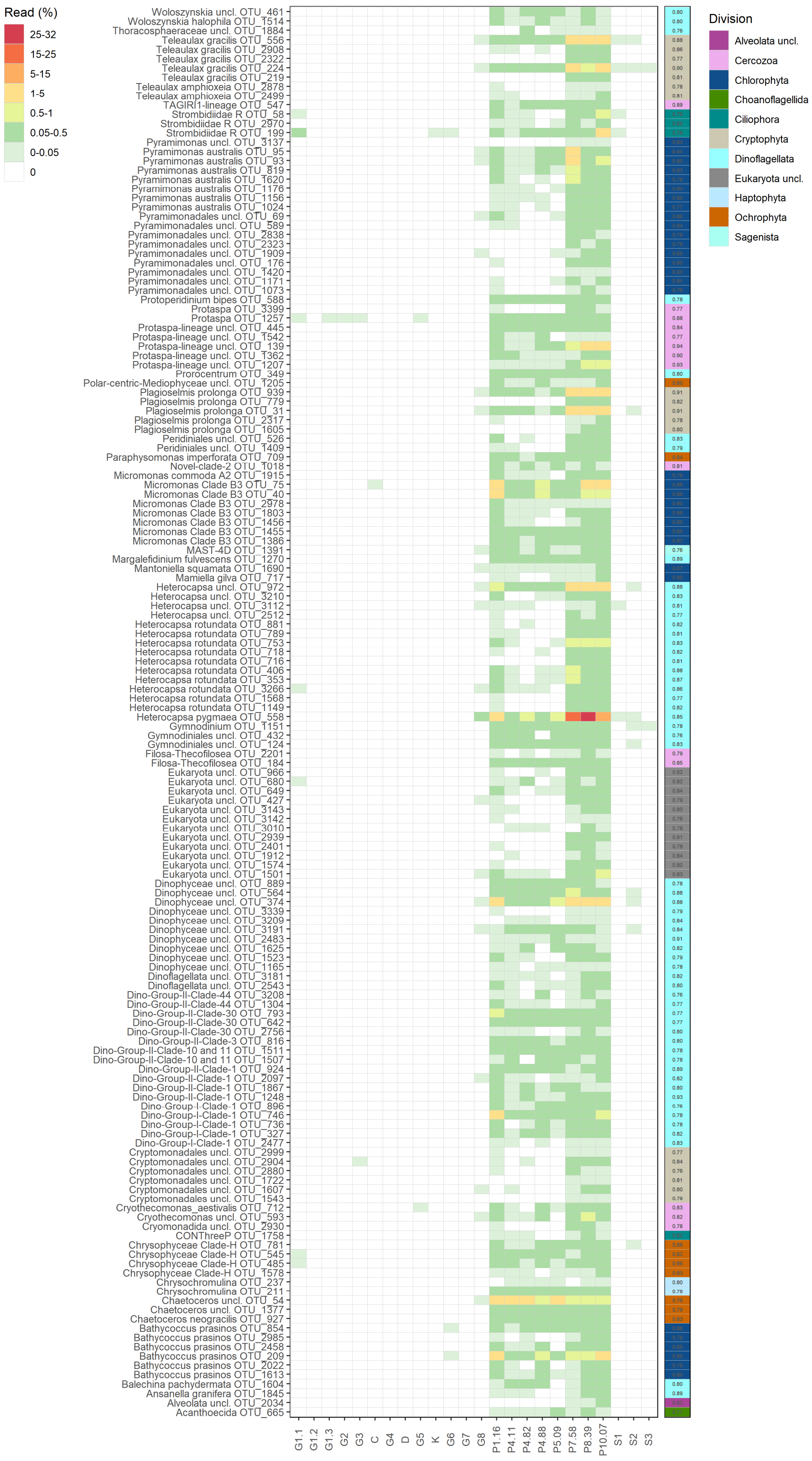

Supplementary Figure S18. Reads proportion (%) of OTUs positively correlated to salinity for the small microbial eukaryote fraction in rDNA. The color in the right column indicates division and the number correspond to Pearson correlation coefficient.

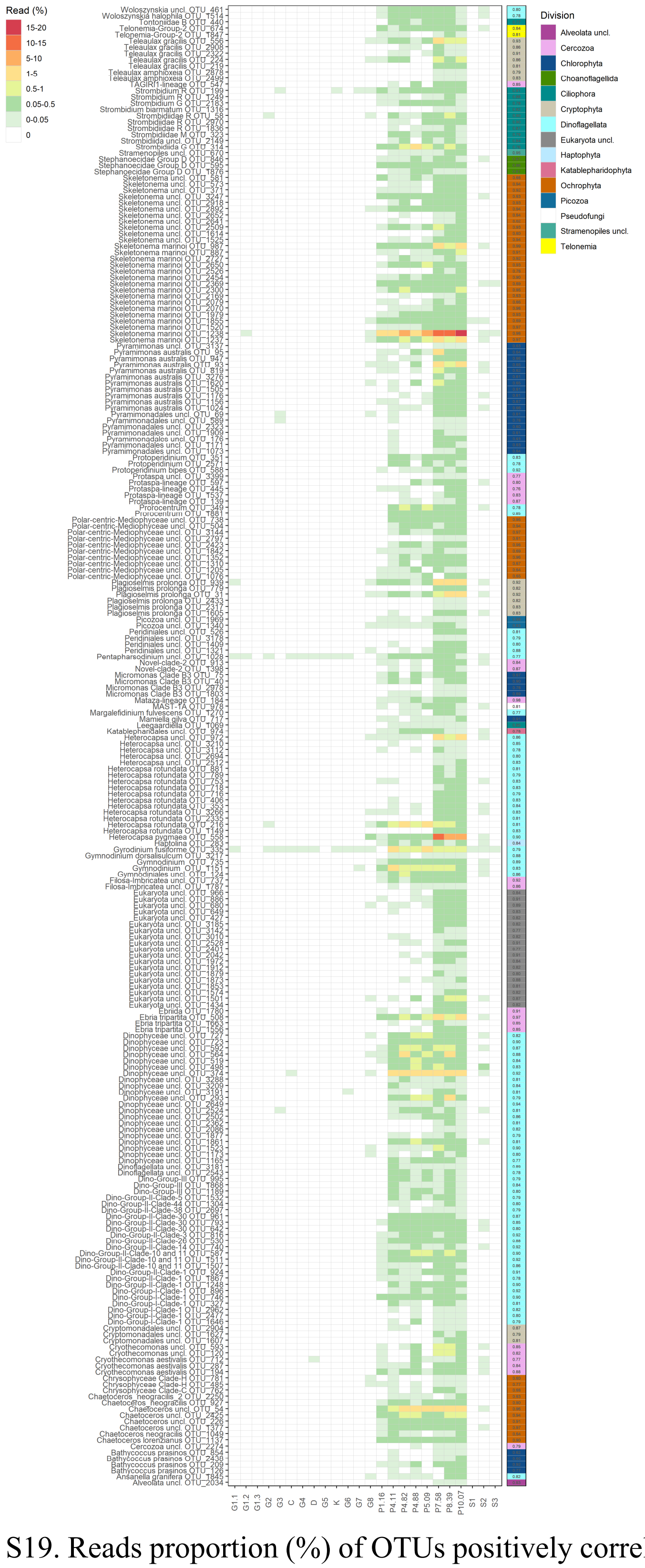

Supplementary Figure S19. Reads proportion (%) of OTUs positively correlated to salinity for the large microbial eukaryote fraction in rDNA. The color in the right column indicates division and the number correspond to Pearson correlation coefficient.

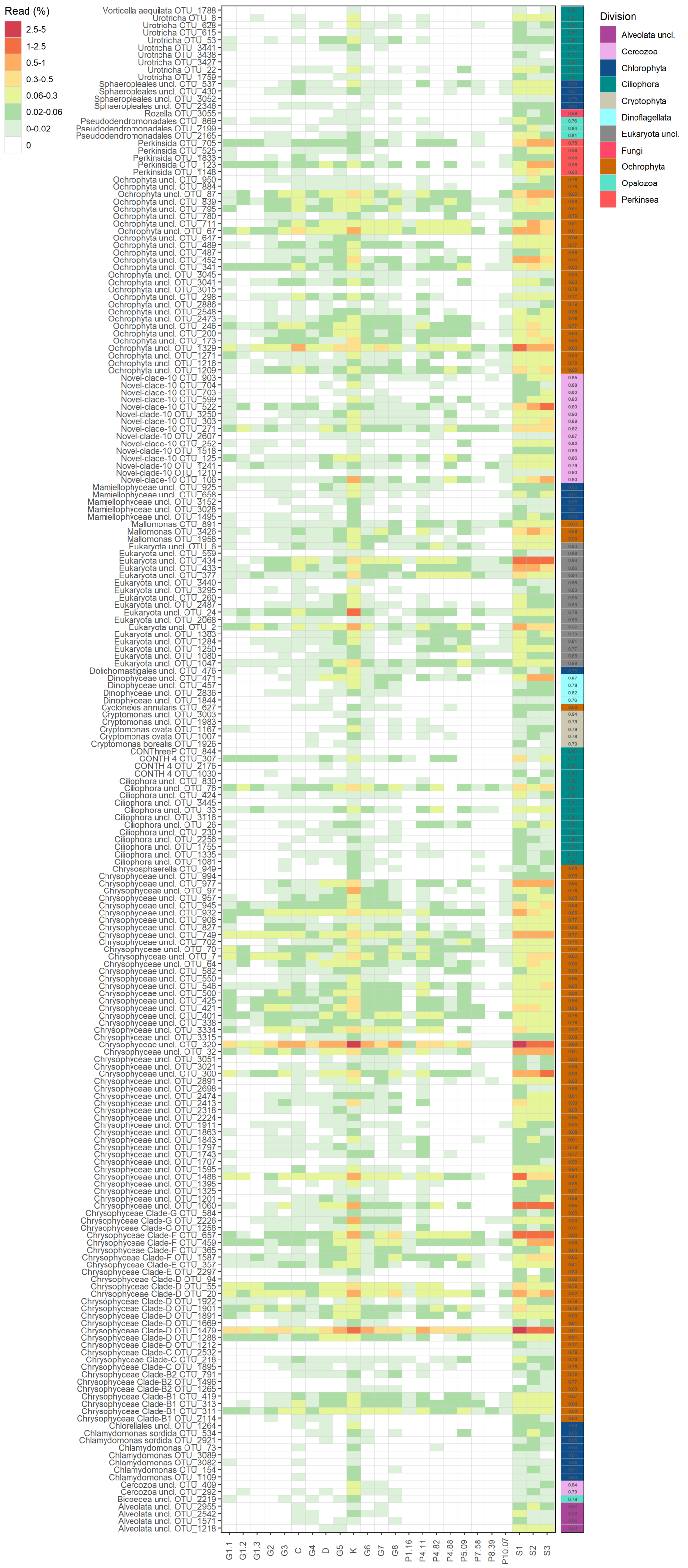

Supplementary Figure S20. Reads proportion (%) of OTUs positively correlated to dissolved organic carbon for the small microbial eukaryote fraction in rDNA. The color in the right column indicates division and the number correspond to Pearson correlation coefficient.

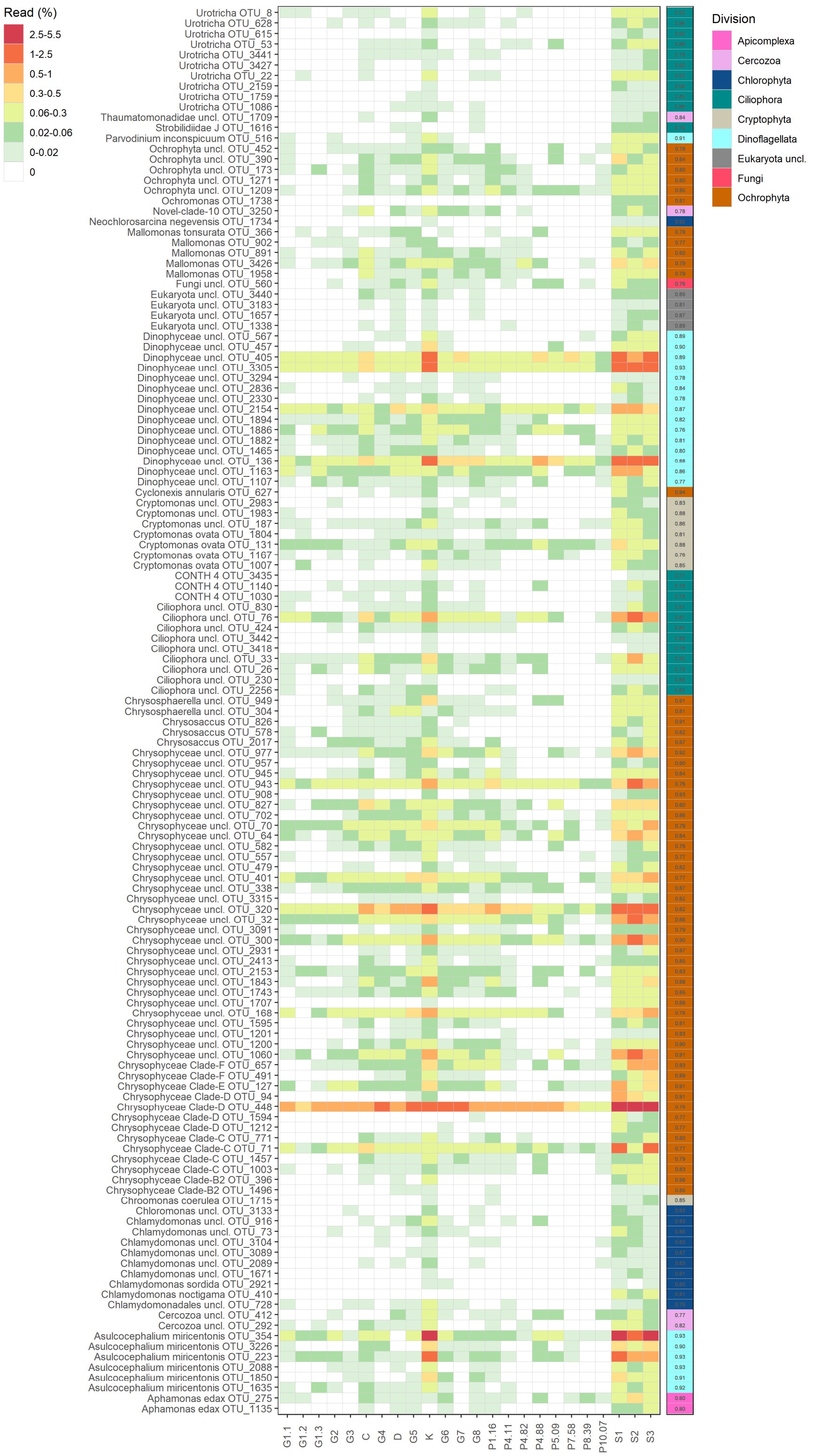

Supplementary Figure S21. Reads proportion (%) of OTUs positively correlated to dissolved organic carbon for the large microbial eukaryote fraction in rDNA. The color in the right column indicates division and the number correspond to Pearson correlation coefficient.

Supplementary Table S1. Amplicon filtration volume (mL).

Site	Amplicon filtration volume
**G1.1**	750
**G1.2**	730
**G1.3**	570
**G2**	665
**G3**	790
**C**	550
**G4**	530
**D**	570
**G5**	520
**K**	220
**G6**	530
**G7**	530
**G8**	620
**P1.16**	1180
**P4.11**	770
**P4.82**	850
**P4.88**	750
**P5.09**	750
**P7.58**	820
**P8.39**	840
**P10.07**	870
**S1**	200
**S2**	220
**S3**	160

Supplementary Table S2. Physicochemical characteristics of each sampling site. In the plume, the salinity was measured in situ at the beginning of the sampling and was verified in each water sample. Note: pH, temperature and dissolved oxygen were only measured in situ.

**Location**	**Site**	**pH**	**Temperature (°C)**	**Dissolved oxygen (%)**	**Salinity (ppt)**	
					in situ	**in sample**
**Great Whale River**	G1	6.63	19.48	103.83	0.013	-
	G2	6.92	18.23	108.38	0.013	-
	G3	6.97	18.42	105.52	0.013	-
	G4	7.25	17.86	112.83	0.013	-
	G5	7.3	17.8	111.42	0.013	-
	G6	7.57	18.11	115.1	0.014	-
	G7	7.22	16.98	123.36	0.013	-
	G8	7.14	16.68	108.66	0.016	-
**Coats River**	C	7.08	16.55	108.18	0.015	-
**Denys River**	D	7.66	16.3	106.14	0.014	-
**Kwakwatanikapistikw River**	K	7.55	15.78	102.39	0.024	-
**Sasapimakwananistikw River**	S1	7.66	16.3	106.14	0.026	-
	S2	7.51	16.95	107.26	0.027	-
	S3	7.42	11.65	98.67	0.027	-
**Plume**	P1	7.5	16.93	NA	5.7	4.82
						5.09
						4.11
	P2	7.66	14.98	110.30	10.21	10.07
						8.39
						7.58
	P3	7.73	13.89	98.49	15.98	1.16
						4.88
						14.86[a]

[a]No amplicons analyses.

Supplementary Table S3. Alpha diversity of the bacterioplankton.

Site	Observed Richness				Shannon			
	rDNA		rRNA		rDNA		rRNA	
	Small	Large	Small	Large	Small	Large	Small	Large
**G1.1**	1166.77	2189.76	1614.87	2483.94	4.29	5.55	5.26	5.77
**G1.2**	1314.59	1587.2	1493.09	1941.54	5.04	5.1	5.21	5.26
**G1.3**	1294.04	1661.74	806.34	1290.64	5.03	5.17	4.72	4.91
**G2**	1278.74	1782.5	1736.19	2500.45	4.95	5.25	5.36	5.82
**G3**	1515.65	2021.84	2103.81	2958.2	5.05	5.33	5.42	6.11
**C**	1295.04	1525.03	1700.07	2202.97	5.09	5.16	5.23	5.45
**G4**	1317.58	2088.84	1695.25	2616.06	5.01	5.52	5.33	5.83
**D**	1501.39	2050.13	1839.9	2285.3	5.21	5.44	5.53	5.66
**G5**	1512.73	1935.24	1867.31	2465.71	5.08	5.31	5.47	5.78
**K**	1097.5	1813.43	1528.45	2137.94	4.73	4.98	4.94	5.48
**G6**	1500.59	1714.68	1892.3	2431.66	5.08	5.22	5.5	5.75
**G7**	1417.36	1885.98	1762.56	2451.81	5.12	5.34	5.46	5.76
**G8**	1475.05	1885.19	1813.71	2507.41	5.08	5.28	5.44	5.78
**P1.16**	1577.86	2169.68	1268.82	2479.27	5.47	5.6	5.06	5.82
**P4.11**	1526.46	1803.25	1857.52	2625.11	5.21	5.35	5.53	6.08
**P4.82**	1427.57	1908.1	911	1179.73	5.19	5.54	4.68	4.83
**P4.88**	1486.64	1951.68	723.69	2418.52	5.33	5.58	3.97	6.17
**P5.09**	1393.86	2007.13	1792.34	2399.7	5.23	5.6	5.7	5.98
**P7.58**	1586.55	2006.97	1728.7	2352.31	5.46	5.77	5.8	6.11
**P8.39**	1520.64	1977.5	1638.58	2189.01	5.46	5.69	5.74	6.02
**P10.07**	1619.46	2056.9	1514.69	2164.66	5.49	5.73	5.38	5.77
**S1**	1242.94	2255.99	2069.34	2745.14	4.53	5.3	5.36	5.73
**S2**	1245.43	1853.37	2043.43	2206.06	4.46	4.99	5.11	5.26
**S3**	1264.31	1979.22	2002.28	2331.15	4.49	5.01	5.04	5.44

Supplementary Table S4. Alpha diversity of the microbial eukaryotes.

Site	Observed Richness				Shannon			
	rDNA		rRNA		rDNA		rRNA	
	Small	Large	Small	Large	Small	Large	Small	Large
**G1.1**	779.15	650.43	560.62	673.25	5.85	5.52	5.14	5.4
**G1.2**	704.6	604.63	447.82	154.03	5.57	5.24	4.55	2.32
**G1.3**	724.67	650.4	24.03	34.7	5.6	5.47	2.43	1.8
**G2**	721.99	676.4	297	618.12	5.62	5.5	3.96	5.13
**G3**	746.05	722.91	641.11	676.37	5.6	5.64	5.18	5.31
**C**	541.4	736.21	724.18	508.22	5.01	5.61	5.85	3.84
**G4**	541.61	688.97	713.9	540.13	4.27	5.52	5.62	4.43
**D**	709.1	738.83	657.65	760.24	5.62	5.74	5.35	5.81
**G5**	797.22	845.3	753.7	787.09	5.86	5.98	5.62	5.87
**K**	642.72	554.53	549.57	524.15	5.67	5.36	5.5	5.37
**G6**	682.91	797.59	513.13	760.6	4.99	5.77	4.16	5.77
**G7**	654.65	782.61	661.1	629.47	5.04	5.78	5.23	5.14
**G8**	751.12	779.77	368.61	607.67	5.64	5.89	3.32	4.59
**P1.16**	543.45	752.33	362.15	630.82	4.86	5.66	4.37	4.84
**P4.11**	866.24	693.16	801.66	806.99	5.82	5.27	5.8	5.79
**P4.82**	808.91	645.04	155.96	39.93	5.62	5.25	4.26	2.49
**P4.88**	766.89	732.56	12.35	731.66	5.47	5.64	1.75	5.3
**P5.09**	815.24	620.96	696.42	406.63	5.58	5.35	5.44	4.16
**P7.58**	575.22	646.8	614.1	552.69	4.59	5.14	4.98	4.22
**P8.39**	513.95	615.65	604.33	530	4.21	5.13	4.97	4.15
**P10.07**	546.18	508.29	304.92	392.52	4.71	4.44	3.96	3.55
**S1**	609.92	574.2	533.3	514.75	5.53	5.26	5.37	4.87
**S2**	655.73	603.36	570.68	621.47	5.74	5.44	5.5	5.59
**S3**	684.8	598.52	572.4	569.21	5.82	5.5	5.42	5.38
